# RNF115 plays dual roles in innate antiviral responses by catalyzing distinct ubiquitination of MAVS and MITA

**DOI:** 10.1038/s41467-020-19318-3

**Published:** 2020-11-02

**Authors:** Zhi-Dong Zhang, Tian-Chen Xiong, Shu-Qi Yao, Ming-Cong Wei, Ming Chen, Dandan Lin, Bo Zhong

**Affiliations:** 1 Department of Virology, College of Life Sciences, Zhongnan Hospital of Wuhan University, 430071 Wuhan, China; 2 Department of Gastrointestinal Surgery, Zhongnan Hospital of Wuhan University, 430071 Wuhan, China; 3 Department of Immunology, Medical Research Institute and Frontier Science Center for Immunology and Metabolism, Wuhan University, 430072 Wuhan, China; 4 Department of Blood Transfusion, Zhongnan Hospital of Wuhan University, 430071 Wuhan, China; 5 Cancer Center, Renmin Hospital of Wuhan University, 430060 Wuhan, China

**Keywords:** Infection, Viral infection, Pattern recognition receptors, Signal transduction

## Abstract

MAVS and MITA are essential adaptor proteins mediating innate antiviral immune responses against RNA and DNA viruses, respectively. Here we show that RNF115 plays dual roles in response to RNA or DNA virus infections by catalyzing distinct types of ubiquitination of MAVS and MITA at different phases of viral infection. RNF115 constitutively interacts with and induces K48-linked ubiquitination and proteasomal degradation of homeostatic MAVS in uninfected cells, whereas associates with and catalyzes K63-linked ubiquitination of MITA after HSV-1 infection. Consistently, the protein levels of MAVS are substantially increased in *Rnf115*^−/−^ organs or cells without viral infection, and HSV-1-induced aggregation of MITA is impaired in *Rnf115*^−/−^ cells compared to the wild-type counterparts. Consequently, the *Rnf115*^−/−^ mice exhibit hypo- and hyper-sensitivity to EMCV and HSV-1 infection, respectively. These findings highlight dual regulation of cellular antiviral responses by RNF115-mediated ubiquitination of MAVS and MITA and contribute to our understanding of innate immune signaling.

## Abstract (web summary)

MAVS and MITA are adapter proteins that play distinct roles in the context of the host response to RNA and DNA viruses, respectively. Here the authors implicate RNF115 in dual temporal and spatial mechanisms of interacting and catalyzing distinct ubiquitination of MAVS and MITA to modulate RNA and DNA antiviral immune responses.

## Introduction

Detection of pathogen-associated molecular patterns (PAMPs) by pattern-recognition receptors (PRRs) represents the first step to initiate immune responses against invading pathogens^1^. Viral RNAs and DNAs generated during viral infection and replication are classical PAMPs that are detected by various PRRs including Toll-like receptors (TLRs), RIG-I-like receptors (RLRs) and cytoplasmic DNA sensors^2–4^. Upon binding to the PAMPs, the PRRs either directly recruit and activate downstream adaptor proteins or catalyze the synthesis of ligands to indirectly activate downstream adaptors. For example, RIG-I binds to dsRNA or 5′ triphosphorylated ssRNA and undergoes conformational change to recruit and induce oligomerization of the adaptor protein MAVS (also known as VISA, IPS-1, and Cardif)^5–9^. In contrast, the cyclic GMP-AMP (cGAMP) synthase (cGAS) binds to dsDNA or DNA–RNA hybrid and catalyzes the synthesis of cGAMP that binds to and induces oligomerization of the adaptor protein MITA (also known as STING and ERIS)^10–15^. Aggregated MAVS and MITA further recruit kinases such TBK1 and IKKε or IKKα/β/γ complex to activate transcription factors IRF3 and NF-κB. These transcription factors enter into nucleus and promote transcription of a large array of downstream genes whose products collaborate to elicit cellular antiviral responses.

The activity and availability of MAVS are strictly regulated by ubiquitination and deubiquitination^16,17^. Upon viral infection, the E3 ligase TRIM31 catalyzes K63-linked ubiquitination and promotes aggregation and activation of MAVS which is prevented by the Otubain family deubiquitinating enzyme YOD1, and TRIM21 induces K27-linked ubiquitination of MAVS to promote the recruitment of TBK1 to MAVS^18–20^. Multiple E3s including AIP4, RNF5, MARCH5, VHL, RNF125, and Smurf1/2 have been reported to induce K48-linked ubiquitination and proteasomal degradation of MAVS to turn down excessive immune responses after viral infection^21–27^, which is counteracted by the deubiquitinating enzyme OTUD4^28^. In addition, viral infection induces K27-linked ubiquitination and autophagic degradation of MAVS by MARCH8 and RNF34 in a NDP52-dependent manner^29,30^. However, whether and how the homeostatic MAVS is regulated through ubiquitination in resting cells is much less understood. We have observed that the levels of MAVS protein remain unchanged until 12 h after cycloheximide (CHX) treatment without viral infection^28^, whereas MG132 treatment substantially upregulates the levels of MAVS and leads to accumulation of ubiquitinaited MAVS (described below), indicating that MAVS is heavily regulated by ubiquitination or deubiquitination in unstimulated conditions and the responsible E3 ligase(s) or deubiquitinating enzyme(s) remain to be identified.

Similar to MAVS, ubiquitin modification of MITA is essential for its stability and activity^16^. In unstimulated cells, TOLLIP associates with MITA and prevent its degradation through lysosome, although whether such a regulation depends on ubiquitination of MITA is unclear^31^. The E3 ligases RNF5 and TRIM29 catalyze K48-linked ubiquitination and proteasomal degradation of MITA after viral infection, which is counteracted by USP18 and USP20^32–36^. AMFR catalyzes and USP13 removes K27-linked ubiquitin chains from MITA^37,38^, thereby positively and negatively regulating the recruitment of TBK1 to phosphorylate IRF3, respectively. It has been recognized that K63-linked ubiquitination of MITA is essential for its activation, as removal of such a modification by USP49 impairs the phosphorylation and aggregation of MITA and the recruitment of TBK^39^. However, it is of debate about the E3s responsible for K63-linked ubiquitination of MITA. Although it has been reported that TRIM56 and TRIM32 promote K63-linked ubiquitination of MITA when overexpressed in cells^40,41^, subsequent gene knockout studies have shown that knockout of either of them did not affect ubiquitination of MITA after viral infection or cGAMP treatment^42,43^. A more recent study shows that MUL1 mediates K63-linked ubiquitination of MITA in cell lines^44^, which is not proven with in vivo mouse models.

In this study, we have identified the E3 ligase RNF115 as a MAVS-interacting and MITA-interacting protein. RNF115 constitutively catalyzes K48-linked ubiquitination of MAVS and regulates homeostatic MAVS levels in uninfected cells. Furthermore, RNF115 interacts with MITA and promotes K63-linked ubiquitination and aggregation of MITA after HSV-1 infection. Consequently, the *Rnf115*^−/−^ mice exhibit increased and decreased resistance to EMCV or HSV-1 infection compared to the wild-type littermates, respectively. These findings highlight the regulation of cellular immune responses against RNA and DNA viruses by modulating distinct ubiquitination of MAVS and MITA by RNF115.

## Results

### RNF115 interacts with MAVS in uninfected cells

**Fig. 1:**
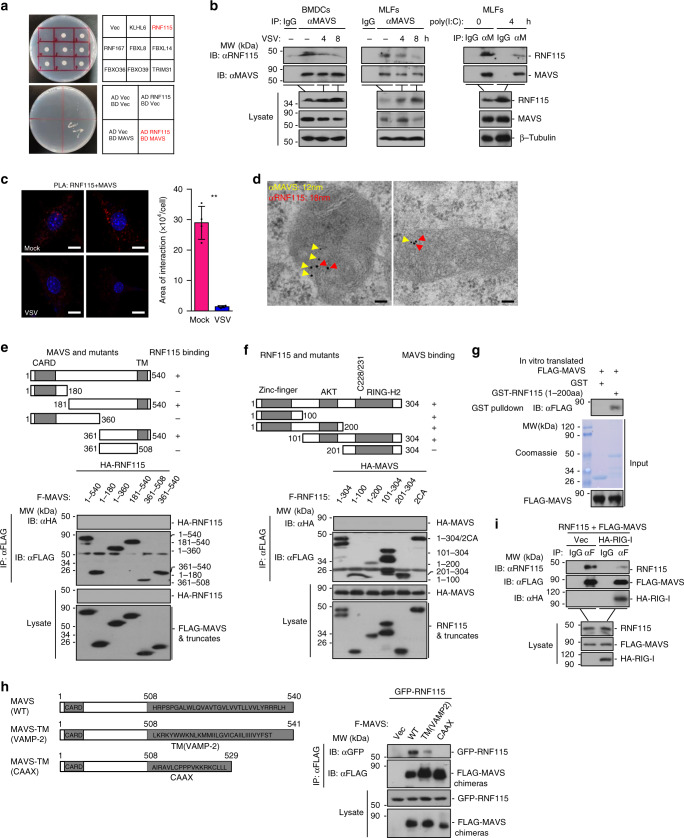
RNF115 constitutively interacts with MAVS in uninfected cells. **a** Yeast-two hybrid assays with AH109 cells that were co-transformed with the indicated plasmids (upper). AH109 cells were co-transformed with pGBT9-MAVS and pGADT7 vector or pGBT9 vector and pGADT7-RNF115 to exclude the self-activation of MAVS and RNF115, respectively (lower). **b** Immunoblot of BMDCs (left panels) and MLFs (middle panels) that were left uninfected or infected with VSV for 4–8 h, or left untransfected or transfected with poly(I:C) (right panels) for 4 h, lysed and immunoprecipitated with a control immunoglobulin G (IgG), or anti-MAVS followed by immunoblot analysis with antibodies to the indicated proteins. **c** In situ PLA assay to examine the colocalization of RNF115 and MAVS in MLFs that were left uninfected or infected with VSV for 6 h (left images). The area of RNF115 and MAVS colocalization was calculated (right graph). RNF115–MAVS complex, red; nuclei, blue. **d** Immunogold staining and electron microscopy analysis of the colocalization of MAVS (smaller beads, 12 nm, yellow arrow head) and RNF115 (larger beads, 18 nm, red arrow head) in MLFs. **e**, **f** Immunoprecipitation (with anti-FLAG) and immunoblot analysis (with anti-FLAG or HA) of HEK293 cells that were transfected with the indicated plasmids, lysed and immunoprecipitated with anti-FLAG. Cell lysate was analyzed by immunoblot with anti-FLAG or anti-HA. **g** GST pulldown assay of GST, GST-RNF115 (1–200aa)(5 μg) and in vitro translated FLAG-MAVS. **h** Immunoprecipitation (with anti-FLAG) and immunoblot analysis (with anti-FLAG or GFP) of HEK293 cells that were transfected with plasmids encoding GFP-RNF115 and FLAG-tagged MAVS or MAVS chimeras. Cell lysate was analyzed by immunoblot with anti-FLAG or anti-GFP. **i** Immunoprecipitation (with anti-FLAG) and immunoblot analysis (with anti-FLAG, HA or RNF115) of HEK293 cells that were transfected with plasmids encoding RNF115, HA-RIG-I, and FLAG-MAVS. Cell lysate was analyzed by immunoblot with anti-FLAG, anti-RNF115, or anti-HA. **P < 0.01 (two-tailed Student’s *t*-test). Scale bars represent 10 μm (**c**) or 200 nm (**d**). Data are representative of three (**a**–**c**) or two (**d**–**i**) independent experiments (graphs show mean ± SD in **c**). Source data are provided as a Source Data file.

MAVS is a critical adaptor protein for cellular antiviral responses against RNA viruses^5–8^. We found that MG132 but not bafilomycin A1 (Baf-A1) treatment led to increased levels of MAVS in human HeLa cell line or in primary mouse lung fibroblasts (MLFs), whereas the mRNA levels of *MAVS* or *Mavs* were not affected by such treatments (Supplementary Fig. 1a). These findings prompted us to speculate that there exist E3 ligases constitutively interacting with MAVS and mediating ubiquitination and degradation of MAVS. We made ~200 pGADT7-E3 constructs and performed yeast-two hybrid screening with pGBT9-MAVS in AH109 cells. These efforts led to the identification of seven E3s as MAVS-interacting protein in yeast cells, of which only RNF115 (also known as BCA2 and Raring 7) was associated with MAVS in transient transfection and coimmunoprecipitation assays and potently inhibited SeV-triggered activation of IFN-β promoter in reporter assays in HEK293 cells (Fig. 1a and Supplementary Fig. 1b, c). RNF115 has been shown highly expressed in invasive breast cancers and genome-wide association studies have identified *RNF115* as a susceptible locus for breast cancer^45–47^. In addition, RNF115 has been shown to restrict HIV-1 replication by promoting degradation of HIV-1 Gag and viral particles or by catalyzing SUMOylation of IκBα^48–50^. Whether and how RNF115 regulates innate antiviral signaling by modulating ubiquitination of MAVS is unknown.

Results from endogenous immunoprecipitation assays suggested that RNF115 was constitutively associated with MAVS without infection, and VSV infection or transfection of poly(I:C) impaired their associations in MLFs or bone marrow-derived dendritic cells (BMDCs) (Fig. 1b). Our proximity ligation assay (PLA) and confocal microscopy imaging confirmed that RNF115 and MAVS were colocalized in uninfected MLFs and their colocalization was diminished after VSV infection (Fig. 1c). Consistent with the notion that MAVS is a mitochondrial protein, our immunogold staining and electron microscopy analysis suggested that RNF115 and MAVS were colocalized on mitochondria (Fig. 1d). Results from domain mapping analysis suggest that the transmembrane domain of MAVS (aa 508–540) was essential for RNF115–MAVS interaction, whereas the N-terminal domain of RNF115 (aa 1–200) was responsible for its interaction with MAVS (Fig. 1e, f). Glutathione S-transferase (GST) pull-down assays revealed a direct interaction between RNF115 and MAVS in vitro (Fig. 1g). To examine whether the mitochondrial localization of MAVS is required for MAVS–RNF115 interaction, we made chimeric MAVS by replacing its transmembrane domain with sequences targeting to ER [MAVS-TM(VAMP-2)] or cell membrane [MAVS-TM(CAAX)] and found that MAVS-TM(VAMP-2) but not MAVS-TM(CAAX) interacted with RNF115 (Fig. 1h)^8^. Taken together, these results suggested that RNF115 constitutively interacts with MAVS and viral infection leads to their disassociation.

We next examined the subcellular localization of RNF115 by cell fractionation and confocal microscopy assays in the presence or absence of viral infections. The results suggested that a portion of RNF115 was found in the mitochondrial fractions and colocalized with mitotracker in MLF cells (Supplementary Fig. 1d, e). However, viral infection did not lead to disappearance of RNF115 from the mitochondrial fractions or mitotracker (Supplementary Fig. 1d, e), suggesting that viral infection-resulted impairment of RNF115 and MAVS association is not due to disassociation of RNF115 from mitochondria. Interestingly, we found that expression of RIG-I impaired RNF115–MAVS but not RNF115–MAVS–TM(VAMP-2) associations (Fig. 1i and Supplementary Fig. 1f), indicating that RIG-I competitively engages MAVS against RNF115 on the mitochondria which might be responsible for the disassociation of RNF115 from MAVS after viral infections.

We noted that RNA virus infection or transfection of poly(I:C) induced upregulation of RNF115 at protein and mRNA levels (Fig. 1b and Supplementary Fig. 1g). The upregulation of *Rnf115* mRNA was completely inhibited by Actinomycin D (Act D) treatment (Supplementary Fig. 1g), indicating that RNA virus infection promotes the transcription but not the stability of *Rnf115* mRNA. However, deficiency of IRF3/7 or p65 did not affect VSV-induced upregulation of *Rnf115* mRNA in MEFs (Supplementary Fig. 1h), suggesting that the classical transcription factors NF-κB or IRF3/7 are dispensable for RNA virus-induced transcription of *Rnf115*. In addition, we further found that transfection of poly(I:C) or infection of VSV upregulated RNF115 protein levels in the presence of Act D or CHX (Supplementary Fig. 1i), indicating that RNA virus infection regulates the expression of RNF115 at translational and posttranslational levels. Collectively, these data strongly suggest that RNA virus-triggered signaling upregulates RNF115 at transcriptional, translational, and posttranslational levels.

### RNF115 deficiency potentiates RNA virus-triggered signaling

Because MAVS is essential for virus infection-induced signaling and interacts with RNF115, we next investigated the role of RNF115 in RNA virus-triggered signaling. We designed two shRNAs targeting RNF115, both of which potently inhibited the expression of RNF115 and significantly upregulated the SeV-induced or VSV-induced expression of *IFNB*, *IFNA4*, and *IL6* (Supplementary Fig. 2a). In addition, knockdown of RNF115 potentiated VSV-induced phosphorylation of TBK1, IκBα, and IRF3 in HeLa cells (Supplementary Fig. 2b). In human CD14^+^ monocyte-derived macrophages, knockdown of RNF115 significantly promoted the expression and production of type I IFNs and phosphorylation of TBK1, IκBα, and IRF3 after VSV infection (Supplementary Fig. 2c–e). These data suggest that RNF115 negatively regulates RNA virus-triggered signaling.

To further investigate the role of RNF115 in antiviral responses, we generated RNF115-deficient mice by CRISPR/Cas9-mediated genome editing (Supplementary Fig. 3a). Sequencing of the mouse *Rnf115* genome suggested a 500 bp deletion in the edited allele, which caused a frame shift and led to an early translational termination of RNF115 (aa 1–67) (Supplementary Fig. 3a, b). The knockout of RNF115 was confirmed by PCR of tail genome DNA and by immunoblot analysis with brain, kidney, heart, lung, spleen, and liver tissues (Supplementary Fig. 3c). The offspring of *Rnf115*^+/−^ breeders conformed to the Mendelian inheritance ratio and the *Rnf115*^−/−^ mice developed and grew normally as the wild-type littermates, indicating that RNF115 is dispensable for the growth and development of mice (Supplementary Fig. 3d). The differentiation of *Rnf115*^−/−^ bone marrow cells into BMDCs and BMDMs was similar to the wild-type counterparts in the presence of GM-CSF and M-CSF cultures, respectively (Supplementary Fig. 3e). The number and composition of immune cells in various organs (including thymus, spleen, and peripheral lymph nodes) were comparable between the *Rnf115*^−/−^ mice and their wild-type littermates at 8-week old (Supplementary Fig. 3f–h), suggesting that RNF115 is not necessary for the development and homeostasis of lymphocytes.

**Fig. 2:**
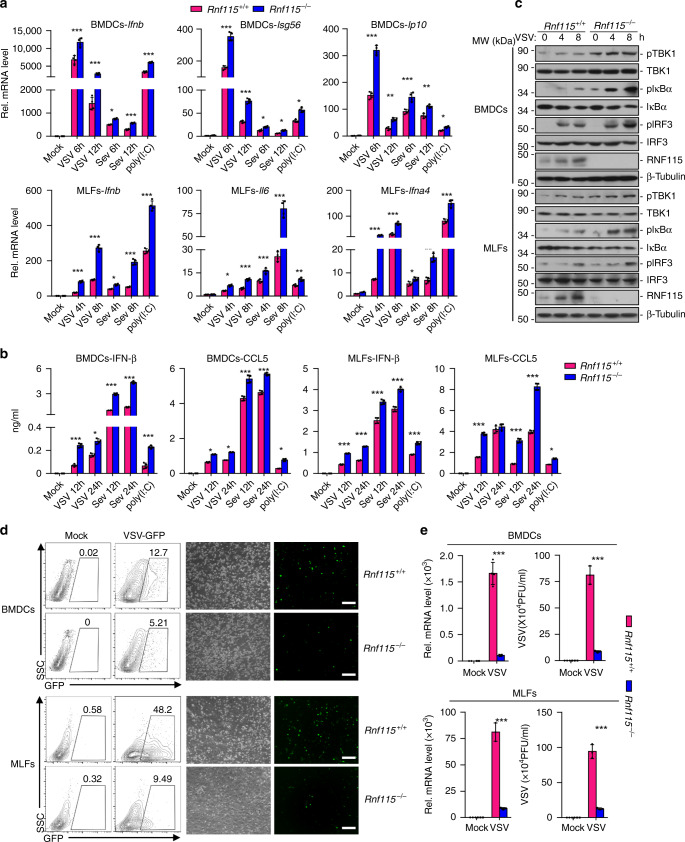
RNF115 deficiency potentiates RNA virus-triggered signaling. **a** qRT-PCR analysis of *Ifnb*, *Isg56*, or *Ip10* mRNA in *Rnf115*^+/+^ and *Rnf115*^−/−^ BMDCs or MLFs infected with VSV or Sev for 0–12 h or transfected with poly(I:C) for 6 h. **b** ELISA analysis of IFN-β and CCL5 in the supernatants of *Rnf115*^+/+^ and *Rnf115*^−/−^ BMDCs or MLFs infected with VSV or SeV for 12–24 h, or transfected with poly(I:C) for 12 h. **c** Immunoblot analysis of total and phosphorylated (p-)IκBα, IRF3, and TBK1, RNF115 and β-Tubulin in *Rnf115*^+/+^ and *Rnf115*^−/−^ BMDCs or MLFs infected with VSV for 0–8 h. **d** Flow cytometric analysis (left flow charts) and microscopy imaging (right images) of the replication of VSV-GFP (MOI = 0.5) in *Rnf115*^+/+^ and *Rnf115*^−/−^ BMDCs or MLFs left uninfected or infected with VSV-GFP for 1 h followed by twice PBS wash and cultured in full medium for 24 h. Numbers adjacent to the outlined areas indicate percentages of GFP^+^ cells. **e** qRT-PCR analysis of VSV-N mRNA in *Rnf115*^+/+^ and *Rnf115*^−/−^ BMDCs or MLFs left uninfected or infected with VSV for 1 h followed by twice PBS wash and cultured in full medium for 24 h (left graphs). Plaque assay of VSV replication in the supernatants of *Rnf115*^+/+^ and *Rnf115*^−/−^ BMDCs or MLFs infected with VSV for 1 h followed by twice PBS wash and cultured in full medium for 48 h (right graphs). *P < 0.05, **P < 0.01, and ***P < 0.001 (two-tailed Student’s *t*-test). Scale bars represent 200 μm. Data are representative of three independent experiments (graphs show mean ± SD in **a**, **b**, **e**). Source data are provided as a Source Data file.

We next examined the effects of RNF115 deficiency on RNA virus-induced expression of downstream genes. The results from qRT-PCR analysis suggested that the expression of *Ifnb*, *Isg56*, and *Ip10* was significantly increased in *Rnf115*^−/−^ BMDCs and MLFs compared to the wild-type counterparts after infection with VSV, SeV, or EMCV, or transfection with poly(I:C) (Fig. 2a and Supplementary Fig. 4a). In addition, *Rnf115*^−/−^ BMDCs or MLFs produced higher levels of IFN-β and CCL5 than did the wild-type counterparts after SeV, VSV, or EMCV infection or transfection of poly(I:C) (Fig. 2b and Supplementary Fig. 4b). Consistent with the gene induction assays, RNF115 deficiency potentiated VSV-, EMCV- or SeV-induced phosphorylation of IRF3, IκBα, and TBK1 in BMDCs and MLFs (Fig. 2c and Supplementary Fig. 4c, d). In addition, the replication of VSV or VSV-GFP was compromised in *Rnf115*^−/−^ BMDCs or MLFs compared to the wild-type counterparts as monitored by the GFP intensities, the expression of VSV-N gene or the VSV titers in the supernatants (Fig. 2d, e). Taken together, these data demonstrate that RNF115 negatively regulates RNA virus-triggered signaling.

### RNF115 deficiency inhibits HSV-1-triggered signaling

**Fig. 3:**
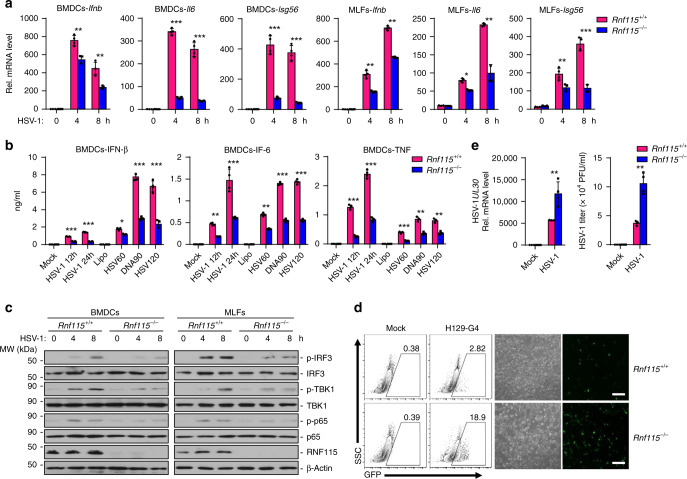
RNF115 deficiency inhibits DNA virus-triggered signaling. **a** qRT-PCR analysis of *Ifnb, Il6,* and *Isg56* mRNA in *Rnf115*^+/+^ and *Rnf115*^−/−^ BMDCs or MLFs infected with HSV-1 for 0–8 h. **b** ELISA analysis of IFN-β, IL6, and TNF in the supernatants of *Rnf115*^+/+^ and *Rnf115*^−/−^ BMDCs infected with HSV-1 for 0–24 h, or transfected with HSV60, DNA90, or HSV120 for 0–6 h. **c** Immunoblot analysis of total and phosphorylated (p-) IRF3, TBK1, P65, and RNF115 and β-Actin in *Rnf115*^+/+^ and *Rnf115*^−/−^ BMDCs or MLFs infected with HSV-1 for 0–8 h. **d** Flow cytometric analysis (left flow charts) and microscopy imaging (right images) of the replication of H129-G4 (MOI = 0.5) in *Rnf115*^+/+^ and *Rnf115*^−/−^ MLFs left uninfected or infected with H129-G4 for 1 h followed by twice PBS wash and cultured in full medium for 24 h. Numbers adjacent to the outlined areas indicate percentages of GFP^+^ MLFs. **e** qRT-PCR analysis of HSV-1 *UL30* mRNA (left graph) and plaque assays (right graph) analyzing HSV-1 titers in *Rnf115*^+/+^ and *Rnf115*^−/−^ MLFs infected with HSV-1 (MOI = 0.5) for 1 h followed by twice PBS wash and cultured in full medium for 24 h. *P < 0.05, **P < 0.01, and ***P < 0.001 (two-tailed Student’s *t*-test). Scale bars represent 200 μm. Data are representative of three independent experiments (graphs show mean ± SD in **a**, **b**, **e**). Source data are provided as a Source Data file.

MAVS is known as an adaptor protein for RNA virus-triggered signaling^51,52^. However, we found that knockdown of RNF115 inhibits HSV-1-, cytoplasmic dsDNA-, or cGAMP-induced expression of *IFNB*, *IL6*, *ISG15*, or *ISG56* in THP-1 cells and HSV-1-induced expression and production of type I IFNs in human CD14^+^ monocyte-derived macrophages (Supplementary Figs. 2c, d and  5a, b). In addition, HSV-1- or transfected dsDNA-induced phosphorylation of IRF3, TBK1, and p65 was substantially restricted by knockdown of RNF115 in THP-1 cells or in human CD14^+^ monocyte-derived macrophages (Supplementary Figs. 2e and 5c), indicating that RNF115 positively regulates DNA virus-triggered immune signaling. Consistently, the expression of *Ifnb*, *Il6*, or *Isg56* and the production of IFN-β, IL-6, and TNF were significantly impaired in primary *Rnf115*^−/−^ MLFs or BMDCs compared to the wild-type counterparts after infection with HSV-1, or transfection with dsDNA ligands or cGAMP (Fig. 3a, b and Supplementary Fig. 6a, b). In addition, HSV-1- or transfected dsDNA-induced phosphorylation of IRF3, TBK1, and p65 was substantially compromised in *Rnf115*^−/−^ BMDCs or MLFs compared to the wild-type controls (Fig. 3c and Supplementary Fig. 6c). Moreover, the replication of HSV-1 or H129-G4 was potentiated in *Rnf115*^−/−^ MLFs or BMDCs compared to the wild-type counterparts as monitored by the expression of HSV-1 *UL30* gene, the HSV-1 titers in the supernatants or the GFP signals of H129-G4 virus (Fig. 3d, e and Supplementary Fig. 6d, e). These data together suggest that RNF115 positively regulates DNA virus-triggered signaling in human cells and multiple types of primary murine cells.

### RNF115 regulates antiviral immune responses in vivo

**Fig. 4:**
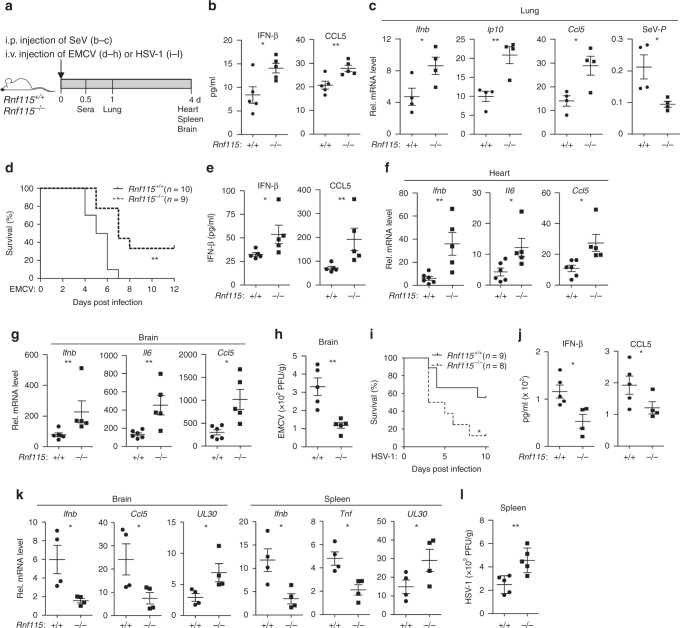
*Rnf115*^−/−^ mice exhibit resistance to EMCV and susceptibility to HSV-1 infection. **a** A scheme of viral infection and analysis of *Rnf115*^+/+^ and *Rnf115*^−/−^ mice. **b**, **c** ELISA analysis of IFN-β and CCL5 in the sera of (**b**) or qRT-PCR analysis of *Ifnb*, *Ip10*, *Ccl5*, and SeV-*P* mRNA in the lungs from (**c**) *Rnf115*^+/+^ (*n* = 5) and *Rnf115*^−/−^ mice (*n* = 5) intraperitoneally injected with SeV for 12 or 24 h, respectively. **d** Survival (Kaplan–Meier curve) of *Rnf115*^+/+^ (*n* = 10) and *Rnf115*^−/−^ mice (*n* = 9) intravenously injected with EMCV (1.8 × 10^6^ PFU per mouse) monitored survival for 12 days. **e** ELISA analysis of IFN-β and CCL5 in the sera of *Rnf115*^+/+^ (*n* = 6) and *Rnf115*^−/−^ mice (*n* = 5) intravenously injected with EMCV (1.8 × 10^6^ PFU per mouse) for 12 h. **f**, **g** qRT-PCR analysis of *Ifnb*, *Il6,* and *Ccl5* mRNA in the hearts (**f**) and brains (**g**) from *Rnf115*^+/+^ (*n* = 6) and *Rnf115*^−/−^ mice (*n* = 5) intravenously injected with EMCV (1.8 × 10^6^ PFU per mouse) for 4 days. **h** Plaque assays analyzing EMCV titers in the brains from *Rnf115*^+/+^ and *Rnf115*^−/−^ mice intraperitoneally injected with EMCV (2 × 10^6^ PFU per mouse) for 4 days. **i** Survival (Kaplan–Meier curve) of *Rnf115*^+/+^ (*n* = 9) and *Rnf115*^−/−^ mice (*n* = 8) intravenously injected with HSV-1 (1 × 10^7^ PFU per mouse) and monitored survival for 10 days. **j** ELISA analysis of IFN-β and CCL5 in the sera of *Rnf115*^+/+^ (*n* = 5) and *Rnf115*^−/−^ mice (*n* = 4) intravenously injected with HSV-1 (2 × 10^7^ PFU per mouse) for 12 h. **k** qRT-PCR analysis of *Ifnb*, *Ccl5*, *Tnf*, and *UL30* mRNA in the brains and spleens from *Rnf115*^+/+^ (*n* = 4) and *Rnf115*^−/−^ mice (*n* = 4) intravenously injected with HSV-1 (2 × 10^7^ PFU per mouse) for 4 days. **l** Plaque assays analyzing HSV-1 titers in the spleens from *Rnf115*^+/+^ and *Rnf115*^−/−^ mice intraperitoneally injected with HSV-1 (2 × 10^7^ PFU per mouse) for 4 days. *P < 0.05, **P < 0.01 (two-tailed Student’s *t*-test or log-rank analysis). Data are of combined three independent experiments (**a**, **f**) or representative of two independent experiments (**b**–**e**, **g**–**i**) (graphs show mean ± SD in **b**–**e**, **g**–**i**). Source data are provided as a Source Data file.

To examine the role of RNF115 in host defense against viral infection in vivo, we infected the *Rnf115*^+/+^ and *Rnf115*^−/−^ mice by intraperitoneal (i.p.) injection of SeV or intravenous (i.v.) injection of EMCV or HSV-1 followed by various analyses (Fig. 4a). The levels of IFN-β and CCL5 protein in the sera and the mRNA levels of *Ifnb*, *Ip10*, and *Ccl5* were significantly increased and the mRNA levels of SeV-P were significantly increased in the lungs of *Rnf115*^−/−^ mice compared to *Rnf115*^+/+^ mice at 12 or 24 h after SeV infection, respectively (Fig. 4b, c). In addition, the *Rnf115*^−/−^ mice showed a later onset of death (day 5 vs. day 4 after EMCV infection) and a higher survival rate than the wild-type littermates (Fig. 4d). The levels of IFN-β and CCL5 were significantly higher in sera from *Rnf115*^−/−^ mice than the wild-type littermates at 12 h after EMCV infection (Fig. 4e). Results from qRT-PCR analysis showed that the expression of *Ifnb*, *Il6,* and *Ccl5* in the hearts and brains of *Rnf115*^−/−^ mice was higher than that of wild-type littermates at day 4 after EMCV infection (Fig. 4f, g). Consistently, the EMCV titers in brains from *Rnf115*^−/−^ mice were significantly lower than in those from *Rnf115*^+/+^ mice (Fig. 4h). These data suggest that knockout of RNF115 results in resistance to EMCV infection.

In contrast, the *Rnf115*^−/−^ mice exhibited increased susceptibility to sub-lethal HSV-1 infection compared to the wild-type littermates (Fig. 4i). In addition, the levels of IFN-β and CCL5 in the sera and the expression of *Ifnb*, *Ccl5*, or *Tnf* in the brains or spleens were significantly suppressed in the *Rnf115*^−/−^ mice compared to those in *Rnf115*^+/+^ littermates at 12 h or day 4 after HSV-1 infection, respectively (Fig. 4j, k). Consistently, the expression of HSV-1 *UL30* gene and HSV-1 titers were increased in the brains or spleens from *Rnf115*^−/−^ mice compared to the wild-type mice at day 4 after HSV-1 infection (Fig. 4k, l), indicating an indispensable role of RNF115 in immune responses against HSV-1 in vivo. Taken together, these data suggest that RNF115 inhibits and facilitates immune responses against RNA and DNA virus infections, respectively.

### RNF115 functions dependently on its enzyme activity

**Fig. 5:**
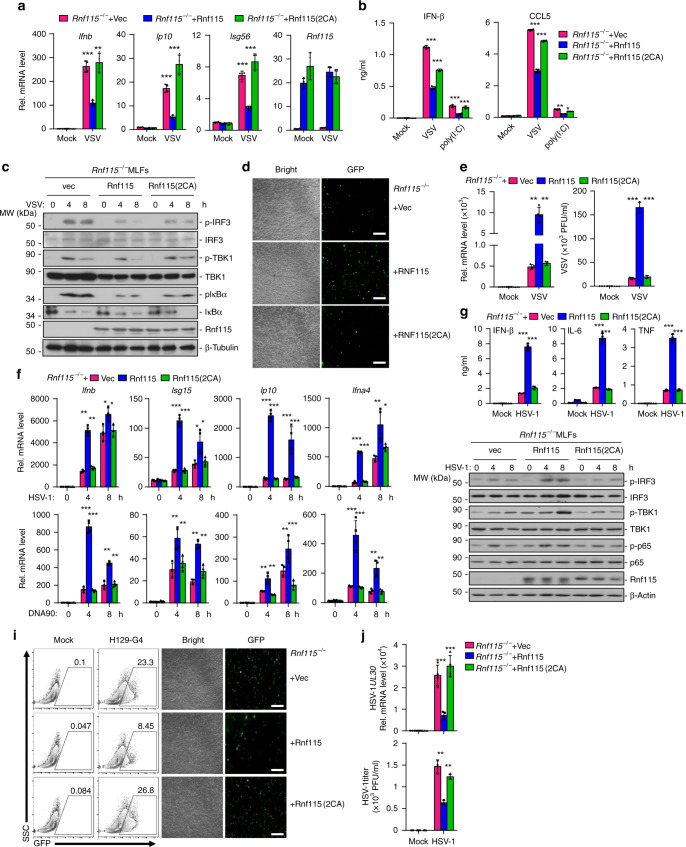
RNF115 functions dependently on its enzyme activity. **a, b** qRT-PCR analysis of *Ifnb*, *Ip10*, *Infa4*, *Isg56,* or *Rnf115* mRNA (**a**) or ELISA analysis of IFN-β and CCL5 (**b**) in *Rnf115*^−/−^ MLFs reconstituted with empty vector (Vec), RNF115, or RNF115(2CA) followed by infection with VSV for 0–6 h (**a**) or by infection with VSV or transfection of poly(I:C) for 12 h (**b**). **c** Immunoblot analysis of total and phosphorylated (p-)IκBα, IRF3, and TBK1, and RNF115, RNF115(2CA), or β-Tubulin in cells obtained in (**a**) infected with VSV for 0–8 h. **d** Fluorescent microscopy imaging of the replication of GFP-VSV in cells obtained in (**a**) infected with VSV-GFP (MOI = 0.5) for 24 h. **e** qRT-PCR analysis of VSV-*N* mRNA (left) or plaque assay of VSV replication in the supernatants of (right) cells obtained in (**a**) left uninfected or infected with VSV for 1 h followed by twice PBS wash and cultured in full medium for 24 or 48 h, respectively. **f**, **g** qRT-PCR analysis of *Ifnb*, *Ip10*, *Infa4*, *Isg56*, or *Rnf115* mRNA (**f**) or ELISA analysis of IFN-β, IL-6, and TNF (**g**) in cells obtained in (**a**) that were infected with HSV-1 or transfected with DNA90 for 0–8 h. **h** Immunoblot analysis of total and phosphorylated (p-)IκBα, IRF3, and TBK1, and RNF115, RNF115(2CA), or β-Tubulin in cells obtained in (**a**) infected with HSV-1 for 0–8 h. **i** Flow cytometric analysis (left graphs) and microscopy imaging (right graphs) of the replication of H129-G4 in cells obtained in (**a**) infected with H129-G4 (MOI = 0.5). Numbers adjacent to the outlined areas indicate percentages of GFP^+^ MLFs. **j** qRT-PCR analysis (upper graph) of *UL30* mRNA and plaque assay (lower graph) analyzing HSV-1 titers in the supernatants of cells obtained in (**a**) infected with HSV-1 (MOI = 0.5) for 1 h followed by twice PBS wash and cultured with full medium for 24 h. *P < 0.05, **P < 0.01, and ***P < 0.001 (two-way ANOVA). Scale bars represent 200 μm. Data are representative of two independent experiments (graphs show mean ± SD in **a**, **b**, **e**, **f**, **g**, **j**). Source data are provided as a Source Data file.

Because RNF115 is an E3 ligase, we asked whether the E3 ligase activity was required for its function in cellular immune responses against RNA or DNA viruses. We reconstituted the empty vector, RNF115 and its enzymatic inactive mutant RNF115 (C228A/C231A) [designated as RNF115(2CA)] in *Rnf115*^−/−^ MLFs followed by infection with VSV or HSV-1 or transfection with poly(I:C) or dsDNA. Results from qRT-PCR and ELISA assays suggested that VSV- or cytoplasmic poly(I:C)-induced expression of *Ifnb*, *Ip10*, or *Isg56*, production of IFN-β and CCL5, or phosphorylation of TBK1, IRF3, or IκBα were inhibited by reconstitution of wild-type RNF115 but not RNF115(2CA) (Fig. 5a–c). Consistently with these observations, reconstitution of RNF115 but not RNF115(2CA) in *Rnf115*^−/−^ MLFs substantially promoted the replication of VSV or VSV-GFP (Fig. 5d, e). Conversely, HSV-1- or cytoplasmic dsDNA-induced expression of *Ifnb*, *Ip10*, or *Isg15*, production of IFN-β, IL-6, and TNF, or phosphorylation of TBK1, IRF3, or p65 were restored by reconstitution of wild-type RNF115 but not RNF115(2CA) (Fig. 5f–h). The replication of HSV-1 or H129-G4 was compromised in *Rnf115*^−/−^ MLFs reconstituted with RNF115 but not RNF115(2CA) as monitored by the expression of viral genes and titers or by the GFP signals (Fig. 5i, j). These data collectively suggest that RNF115-mediated regulation of cellular antiviral responses requires its E3 ligase enzyme activity.

### RNF115 mediates ubiquitination and degradation of MAVS

**Fig. 6:**
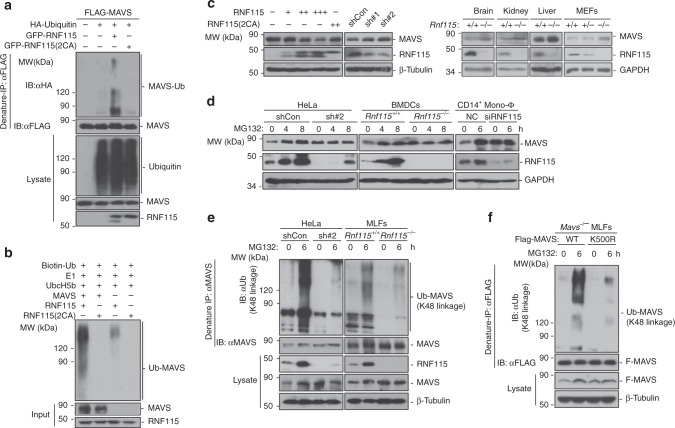
RNF115 mediates K48-linked ubiquitination and proteasomal degradation of homeostatic MAVS. **a** Denature-immunoprecipitation (Denature-IP) (with anti-FLAG) and immunoblot analysis (with anti-FLAG, anti-HA, or anti-GFP) of HEK293 cells transfected with plasmids encoding FLAG-MAVS, HA-Ubiquitin, and empty vector, GFP-RNF115, or GFP-RNF115(2CA) for 24 h. **b** In vitro ubiquitination analysis of ubiquitin-modified MAVS. MAVS, RNF115, and RNF115(2CA) were translated in vitro, Biotin-Ub, E1, and UbcH5 were added for ubiquitination assays. Ubiquitin-conjugated MAVS was detected by immunoblot with HRP–streptavidin (upper panel). The proteins in the input were detected by immunoblot with the indicated antibodies (lower panels). **c** Immunoblot of HeLa cells that were transfected with plasmids encoding RNF115 (0, 200, 400, 600 ng, respectively) and RNF115(2CA) (400 ng) for 24 h (upper left panels), or transfected with shRNA(Con, shRNF115#1 or shRNF115#2) for 36 h (upper right panels), lysed and analyzed by immunoblot with anti-MAVS, RNF115, and β-Tubulin. Immunoblot analysis of RNF115, MAVS, and GAPDH in brains, kidneys, livers from *Rnf115*^+/+^ and *Rnf115*^−/−^ mice, or in *Rnf115*^+/+^, *Rnf115*^+/−^, and *Rnf115*^−/−^ MEFs (lower panels). **d** Immunoblot analysis of MAVS, RNF115, and GAPDH in HeLa cells that were transfected with shCon or shRNF115#2 for 36 h, in *Rnf115*^+/+^ and *Rnf115*^−/−^ MLFs treated with MG132 for 0–8 h or in human CD14^+^ monocytes-derived macrophages transfected with control siRNA (NC) or siRNF115 followed by treatment of MG132 for 0–6 h. **e** Denature-IP (with anti-MAVS) and immunoblot analysis (with anti-K48 linkage polyubiquitin, anti-MAVS, anti-RNF115, or anti-GAPDH) in HeLa cells that were transfected with shCon and shRNF115#2 for 36 h or in *Rnf115*^+/+^ and *Rnf115*^−/−^ MLFs treated by MG132 for 0–6 h. **f** Denature-IP (with anti-FLAG) and immunoblot analysis (with anti-K48 linkage polyubiquitin, anti-FLAG, or anti-GAPDH) of *Mavs*^−/−^ MLFs reconstituted with MAVS or MAVS(K500R) treated with MG132 for 0–6 h. Data are representative of three (**a**, **b**) or two (**c**–**f**) independent experiments. Source data are provided as a Source Data file.

Since RNF115 interacted with MAVS and the E3 activity was required for its regulation of cellular antiviral responses against RNA viruses, we investigated whether RNF115 functions by conjugating poly-ubiquitin chains to MAVS. Expectedly, RNF115 but not RNF115(2CA) catalyzed ubiquitination of MAVS in cells or in vitro (Fig. 6a, b). In addition, we found that overexpression of RNF115 decreased the protein levels of MAVS in a dose-dependent manner and knockdown of RNF115 substantially increased the protein levels of MAVS in HeLa, HEK293, or THP-1 cells without affecting the mRNA levels of *MAVS* (Fig. 6c and Supplementary Fig. 7a). Interestingly, RNF115 catalyzed ubiquitination of MAVS and downregulated the protein levels of MAVS but not MAVS-TM(VAMP2) (Supplementary Fig. 7b, c), indicating that a degradative role of RNF115 for MAVS on mitochondria. In addition, the protein levels of MAVS were substantially increased in RNF115 knockout primary murine cells or organs, whereas the mRNA levels of *Mavs* were similar between RNF115-deficient and sufficient cells or organs (Fig. 6c and Supplementary Fig. 7d, e), suggesting that RNF115 regulates the stability of MAVS protein under uninfected homeostatic conditions.

We next examined the stability of homeostatic MAVS and found that CHX treatment did not decrease the protein levels of MAVS but resulted in degradation of RNF115 which was inhibited by MG132 treatment (Supplementary Fig. 7f), indicating proteasome-dependent rapid turnover of homeostatic RNF115 in uninfected cells. In this context, we observed that MG132 treatment led to accumulation of RNF115 in cells (Fig. 6d). In addition, MG132 treatment also resulted in accumulation of MAVS in HeLa cells transfected with control shRNA, in *Rnf115*^+/+^ BMDCs or in human CD14^+^ monocyte-derived macrophages transfected with control siRNA but not in HeLa cells transfected with control shRNF115, in *Rnf115*^−/−^ BMDCs or in human CD14^+^ monocyte-derived macrophages transfected with siRNF115 (Fig. 6d). MG132 inhibits proteasome which recognizes and degrades substrates modified by K48-linked ubiquitin chains. In support of this notion, MG132 substantially increased K48-linked ubiquitination of MAVS, which was almost abolished by knockdown of RNF115 in HeLa cells or knockout of RNF115 in MLFs (Fig. 6e), indicating that RNF115 is the major E3 ligase that mediates K48-linked ubiquitination and degradation of homeostatic MAVS.

We next mapped the lysine residue(s) on MAVS to which ubiquitin is attached by RNF115 and found eight putative lysine target residues, K7, K10, K270, K311, K362, K371, K461, and K500 via website prediction (http://www.phosphosite.org). We generated FLAG-MAVS mutants in which each of these lysine residues was replaced with an arginine (R) residue and found that mutation of K500 to arginine abolished RNF115-mediated ubiquitination of MAVS in cells or in vivo (Supplementary Fig. 7g, h). We reconstituted the FLAG-tagged MAVS or MAVS(K500R) into *Mavs*^−/−^ MLFs followed by MG132 treatment and ubiquitination assays. The results showed that MG132 treatment increased the protein levels of and the K48-linked ubiquitination of MAVS but not MAVS(K500R) (Fig. 6f). Together, these data demonstrate that RNF115 constitutively catalyzes K48-linked ubiquitination on Lys500 of homeostatic MAVS and induces its protesomal degradation.

### RNF115 interacts with MITA after HSV-1 infection

Because RNF115 positively regulates DNA virus- or cytoplasmic DNA-induced signaling and the E3 ligase activity is required, we speculated that RNF115 might target proteins involved in DNA virus-triggered pathway for ubiquitination. In transient transfection and coimmunoprecipitation experiments, we found that RNF115 was precipitated with MITA and MAVS but not with other molecules involved in RNA or DNA-virus-triggered signaling including RIG-I, cGAS, TBK1, or IRF3 (Supplementary Fig. 8a). Endogenous coimmunoprecipitation assays suggested that RNF115 interacted with MITA in THP-1 cells and MLFs only after HSV-1 infection (Supplementary Fig. 8b). Domain mapping analysis suggested that the transmembrane domains of MITA and the N-terminal Zn-finger and AKT domains of RNF115 were responsible for their association (Supplementary Fig. 8c, d), which is similar to the requirement of domains responsible for RNF115–MAVS associations. Results from cell fractionation and confocal microscopy assays suggested that a portion of RNF115 was localized on the ER which was potentiated by HSV-1 infection (Supplementary Figs. 1e and 8e). These data suggest that HSV-1 infection leads to the accumulation of RNF115 on the ER to interact with MITA.

### RNF115 ubiquitinates and promotes aggregation of MITA

**Fig. 7:**
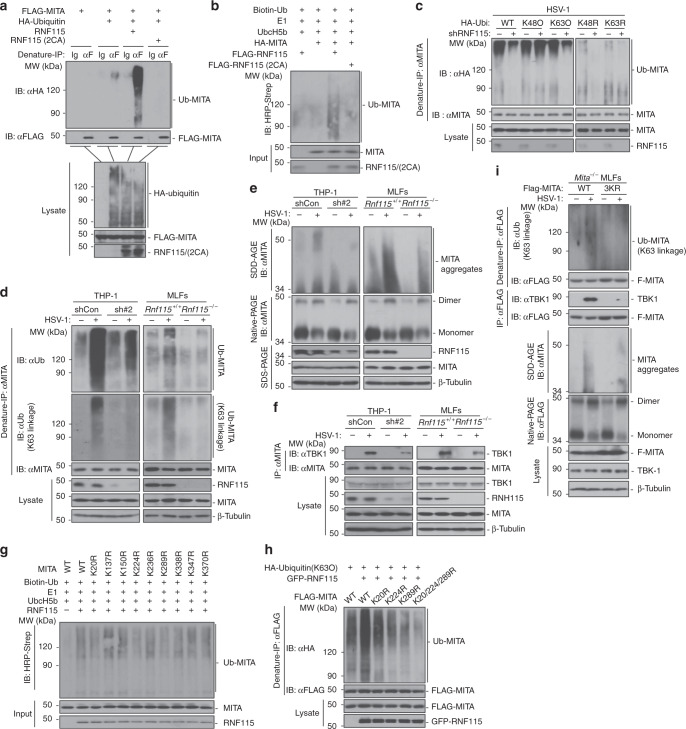
RNF115 interacts with and catalyzes K63-linked ubiquitination of MITA after HSV-1 infection. **a** Denature-IP (with control IgG or anti-FLAG) and immunoblot analysis (with anti-HA, FLAG, or RNF115) of HEK293 cells transfected with the indicated plasmids for 24 h. **b** In vitro ubiquitination analysis of MITA. **c** Denature-IP (with anti-MITA) and immunoblot analysis (with anti-HA, MITA, or RNF115) of THP-1 cells stably shCon or shRNF115#2 that were transfected with plasmids encoding HA-Ubiquitin or HA-Ubiquitin mutants (K48O, K63O, K48R, K63R) followed by HSV-1 infection for 6 h. **d**, **e** Denature-IP (with anti-MITA) and immunoblot analysis (with anti-Ub, K48-linkage polyubiquitin, MITA, RNF115, or β-Tubulin) (**d**) or SDD-AGE analysis of the aggregation of MITA, native-PAGE analysis of the dimerization of MITA and SDS–PAGE analysis of the indicated proteins (**e**) in THP-1 cells stably transfected with shCon or shRNF115#2 or in *Rnf115*^+/+^ and *Rnf115*^−/−^ MLFs infected with HSV-1 for 0–6 h. **f** Immunoprecipitaion (with anti-MITA) and immunoblot analysis (with anti-MITA, TBK1, RNF115, or β-Tubulin) in THP-1 cells stably transfected with shCon and shRNF115#2 (left panels) or in *Rnf115*^+/+^ and *Rnf115*^−/−^ MLFs (right panels) infected with HSV-1 for 0–6 h. **g** In vitro ubiquitination analysis of ubiquitin-modified MITA or MITA mutants. **h** Denature-IP (with anti-FLAG) and immunoblot analysis (with anti-FLAG, HA, or GFP) in HEK293 cells transfected with plasmids encoding FLAG-MITA or mutations, HA-Ubiquitin, empty vector and GFP-RNF115 for 24 h. **i** Denature-IP (with anti-FLAG, upper two panels), immunoprecipitation (with anti-FLAG, middle two panels), and immunoblot analysis (with antiK63 linkage polyubiquitin, FLAG, or TBK1), SDD-AGE analysis (with anti-FLAG or β-Tubulin, middle panels) or native-PAGE analysis (with anti-FLAG) in *Mita*^−/−^ MLFs reconstituted with MITA or MITA(3KR) followed by infection with HSV-1 for 0–6 h. Data are representative of three (**a**–**c**) or two (**d**–**i**) independent experiments. Source data are provided as a Source Data file.

Now that RNF115 interacted with MITA after HSV-1 infection and its enzymatic activity is required for promoting cellular immune responses against HSV-1, we hypothesized that RNF115 may target MITA for ubiquitination. Expectedly, RNF115 but not RNF115(2CA) catalyzed ubiquitination of MITA in cells or in vitro (Fig. 7a, b). To examine the type of RNF115-mediated ubiquitin linkage of MITA, we transfected wild-type ubiquitin, or ubiquitin mutants either retaining a single lysine residue (KO) or retaining all but one lysine residues (KR) in the presence or absence of shRNF115 followed by HSV-1 infection and ubiquitination assays. The results showed that knockdown of RNF115 diminished K63O ubiquitin conjugation but not K63R ubiquitin conjugation to MITA in THP-1 cells after HSV-1 infection (Fig. 7c). Consistently, endogenous ubiquitination analysis revealed that total or K63-linked ubiquitination of MITA was significantly decreased in RNF115-knockdown THP-1 cells or in RNF115-deficient MLFs after infection with HSV-1 (Fig. 7d). These observations collectively suggest that RNF115 targets MITA for K63-linked ubiquitination after HSV-1 infection.

K63-linked ubiquitination of MITA is critical for its oligomerization and the subsequent recruitment of TBK1. Consistently, we found that the aggregation of MITA and the recruitment of TBK1 to MITA were substantially impaired by knockdown of RNF115 in THP-1 cells or by knockout of RNF115 in MLFs after HSV-1 infection (Fig. 7e, f). In contrast, HSV-1-induced dimerization of MITA was not affected by knockdown or knockout of RNF115 (Fig. 7e). Site mapping analysis revealed that mutation of K20, K224, or K289 into arginine residues impaired the ubiquitination by RNF115 and simultaneously mutation of the three residues [MITA(3KR)] abolished RNF115-mediated ubiquitin-K63O conjugation to MITA (Fig. 7g, h). In addition, the aggregation and K63-linked ubiquitination of and the recruitment of TBK1 to MITA(3KR) were substantially impaired compared to the wild-type MITA and the dimerization of MITA(3KR) was comparable to that of wild-type MITA when reconstituted into *Mita*^−/−^ MLFs after HSV-1 infection (Fig. 7i). Knockdown of RNF115 or mutation of the Lys20/224/289 of MITA inhibited the translocation of MITA to the ER–Golgi intermediate compartments (ERGIC) (Supplementary Fig. 8f, g). Expectedly, HSV-1 infection-induced expression of *Ifnb*, *Ccl5*, and *Ip10* was significantly lower in *Mita*^−/−^ MLFs reconstituted with MITA(3KR) than in those reconstituted with MITA (Supplementary Fig. 8h). Taken together, these data demonstrate that RNF115 catalyzes K63-linked polyubiquitination of MITA and enhances the aggregation of MITA and promotes its ERGIC translocation as well as the recruitment of TBK1 after HSV-1 infection.

## Discussion

**Fig. 8:**
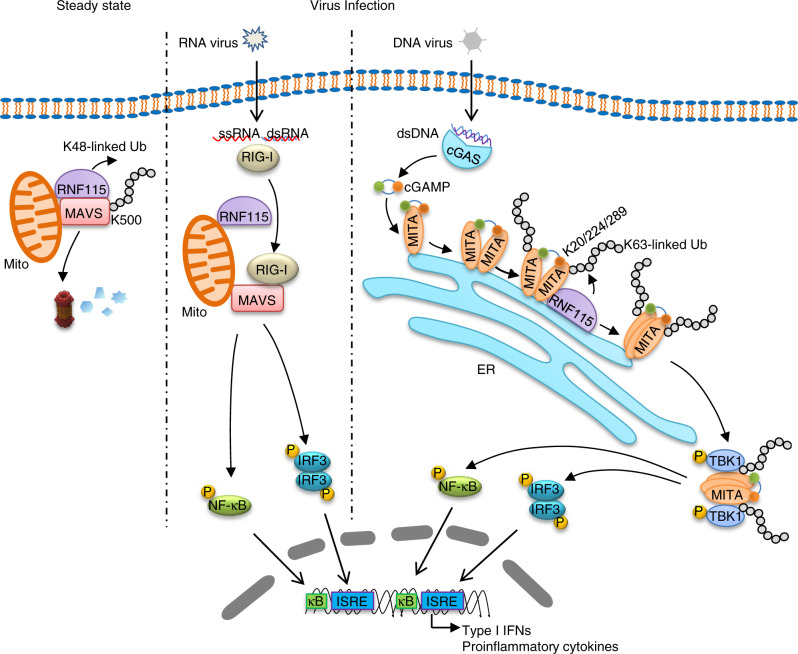
A model on RNF115-mediated regulation of innate antiviral signaling. In unstimulated cells, RNF115 constitutively interacts with and catalyzes K48-linked ubiquitination of MAVS, thereby leading to the degradation of homeostatic MAVS through the proteasome pathways and restricting the basal immune signaling. Upon infection with RNA viruses, RIG-I–MAVS interaction led to RNF115 dissociation from MAVS, thereby eliciting downstream signal transduction. Moreover, infection of DNA viruses induces the accumulation of RNF115 at ER to interact with and catalyze K63-linked ubiquitination of MITA and enhance aggregation of MITA as well as the recruitment of TBK1, which promote the production of type I IFNs and proinflammatory cytokines.

The activity and availability of MAVS and MITA are strictly controlled by ubiquitination and deubiquitination to elicit antiviral immunity and avoid excessive harmful autoimmunity^16^. Although several E3 ligases and deubuqutinating enzymes mediate ubiquitination and deubiquitination of MAVS to modulate its activity after RNA virus infection^17^, the regulation of homeostatic MAVS is unclear. It has been previously shown that PCBP1 interacts with MAVS constitutively before and after SeV infection and recruits AIP4 to mediate degradation of MAVS^53^. However, knockout or knockdown of AIP4 does not lead to accumulation of MAVS in uninfected conditions^26^. In current study, we found that RNF115 constitutively interacted with and induced K48-linked ubiquitination and proteasomal degradation of MAVS in uninfected cells, and SeV infection or cytoplasmic poly(I:C) challenge led to disassociation of RNF115 from MAVS, which was probably due to activated RIG-I by the RNA ligands, suggesting that RNF115 regulates the homeostasis of MAVS protein in steady conditions (Fig. 8). In support of this notion, the protein levels of MAVS were increased in RNF115-knockdown cells or RNF115 knockout cells and organs. Interestingly, however, results from CHX treatment and immunoblot assays revealed that the MAVS protein was stable without infection, whereas the RNF115 protein was rather unstable under steady conditions which was stabilized by MG132 treatment, indicating that RNF115 undergoes rapid turnover in the ubiquitin–proteasome pathway. Such a rapid turnover of RNF115 might prevent constitutive and excessive ubiquitination and degradation of MAVS in uninfected cells. In this context, it has been demonstrated that RNF115 undergoes autoubiquitination that controls its own stability and affects cell migration^54,55^, which might be responsible for the rapid turnover of RNF115 under steady conditions.

In addition to catalyze K48-linked ubiquitination of MAVS in uninfected cells, RNF115 was found to induce K63-linked ubiquitination of MITA after HSV-1 infection or cytoplasmic dsDNA challenge, which promotes the aggregation of and the recruitment of TBK1 to MITA as well as cellular immune responses against DNA viruses. Although previous studies have identified TRIM56 and TRIM32 as enzymes that catalyze K63-linked ubiquitination of MITA^40,41^, gene deletion studies suggest that TRIM56 promotes mono-ubiquitination and activation of cGAS and that TRIM32 deficiency has no effect on HSV-1-triggered signaling^42,43^. Our results have demonstrated RNF115 as a bona fide E3 responsible for virus-triggered K63-linked ubiquitination and activation of MITA. In support of this notion, we observed that (i) the K63-linked ubiquitination and aggregation was substantially impaired in RNF115 knockout cells, (ii) knockout of RNF115 inhibited cGAMP-induced expression of downstream genes, and (iii) MITA(3KR) almost completely lost the aggregation or K63-linked ubiquitination and the ability to recruit TBK1 when reconstituted into *Mita*^−/−^ cells after HSV-1 infection.

It is quite interesting that RNF115 exerts distinct ubiquitin linkage specificity against MAVS and MITA. RNF115 constitutively interacted with MAVS, while its association with MITA depended on HSV-1 infection or transfection of dsDNA. A possible explanation for this is that RNF115 constitutively exerts K48 linkage specificity on mitochondria, while HSV-1 infection alters the ubiquitin linkage specificity of RNF115 by changing its location. In support of this notion, we found that RNF115 catalyzed ubiquitination of and decreased the levels of mitochondrial MAVS but not ER-located MAVS-TM(VAMP-2), though RNF115 interacted with MAVS-TM(VAMP-2). In addition, RNF115 was increased in the ER fraction and decreased in the cytosol fraction after HSV-1 infection, suggesting a translocation of RNF115 from cytosol to ER. How RNF115 is translocated and how its locations determine the ubiquitin linkage specificity require further investigations. Alternatively, the different substrates or pathways determine the distinct linkage specificities of RNF115. It is not surprising, as many E3s exert such functions. For example, TRIM31 exerts K63 linkage specificity to MAVS and K48 linkage specificity to NLRP3, respectively^20,56^, and Nrdp1 catalyzes K33-, K48- or K63-linked ubiquitination of Zap70, MyD88, and TBK1, respectively^57,58^.

In addition to promoting immune responses against DNA viruses, RNF115-mediated dual regulation of MAVS and MITA may provide two layers of protection from host against RNA viruses. Firstly, RNF115 constitutively catalyzed K48-linked ubiquitination on Lys500 of MAVS, and VSV infection or transfection of poly(I:C) disrupted RNF115–MAVS associations, which frees the Lys500 residue that is modified with K63-linked ubiquitination after viral infection in an unknown mechanism for subsequent recruitment and activation of IKKε^59^. Secondly, recent studies have demonstrated that several RNA viruses stimulate the cGAS-STING/MITA pathway through inducing mitochondrial DNA release^60,61^, which may induce the accumulation of RNF115 at ER to interact with and catalyze K63-linked ubiquitination of MITA to amplify the cGAS-STING/MITA signaling and the production of type I IFNs. In this context, we found that VSV or SeV infection increased the availability of RNF115 in the ER fractions or the localization of RNF115 with ER. It should be noted that RNF115 lacks of any transmembrane domains. Previous studies have shown that RNF115 interacts with and stabilized by 14-3-3sigma, a member of the 14-3-3 protein family which is involved in protein trafficking^54^. Whether and how RNF115 is localized to mitochondria or ER in a manner dependent on the 14-3-3 protein family remains to be elucidated.

We noted that RNA virus infection or transfection of poly(I:C) induced upregulation of RNF115 at protein and mRNA levels in HeLa cells, human primary CD14^+^ monocyte-derived macrophages and various primary mouse cells. In contrast, HSV-1 infection or transfection of dsDNA did not upregulate RNF115 in these cells, indicating distinct regulatory mechanisms of *Rnf115* mRNA transcription by RNA and DNA viruses. In this context, the common transcription factors IRF3/7 and p65 downstream of RNA and DNA viruses-triggered immune signaling were dispensable for the transcriptional upregulation of *Rnf115* mRNA after RNA virus infection. Besides transcriptional regulation, RNA virus infection also regulated the RNF115 protein levels post transcription and translation, suggesting transcriptional, translational, and posttranslational levels of RNF115 expression regulated by RNA viruses. Further investigations are required to characterize the key molecules involved in RNA (but not DNA) virus-induced upregulation of RNF115.

Several studies have shown that RNF115 promotes tetherin-dependent and independent HIV-1 restriction^48–50^. It has been shown that cGAS detects retro-transcribed DNA of HIV-1 genome to activate MITA signaling pathway^62^. Our identification of RNF115 as a modifier for K63-linked ubiquitination and aggregation of MITA provided another layer to its restriction of HIV-1, indicating that RNF115 is a target for treatment of HIV-1 infection. Finally, we provide data that highlight the molecular mechanism of RNF115-mediated dual roles in antiviral signaling by regulating homeostatic MAVS in uninfected cells and by promoting the activation of MITA after viral infection.

## Methods

### Mice

The *Rnf115*^−/−^ mice were generated by CRISPR/Cas9-mediated genome editing (GemPharmatech Co. Ltd). In brief, the vectors encoding Cas9 (44758, Addgene) and guide RNAs (5′-TCATTAGGAATCTAAGCATGTGG-3′; 5′-TTGCTGATGTACATAGTTGGTGG-3′) were in vitro transcribed into mRNA and gRNA followed by injection into the fertilized eggs that were transplanted into pseudopregnant mice. The targeted genome of F0 mice was amplified with PCR and sequenced and the chimeras were crossed with wild-type C57BL/6 mice to obtain the *Rnf115*^+/−^ mice. The F1 *Rnf115*^+/−^ mice were further crossed with wild-type C57BL/6 mice for at least three generations. Mice were genotyped by PCR analysis followed by sequencing and the resulted *Rnf115*^+/−^ mice were crossed to generate *Rnf115*^+/+^ and *Rnf115*^−/−^ mice. Age-matched and sex-matched *Rnf115*^+/+^ and *Rnf115*^−/−^ littermates were blindingly randomized into groups for animal studies. The *Mavs*^−/−^ and *Mita*^−/−^ mice were previously described and kindly provided by Dr. Hong-Bing Shu (Wuhan University)^63,64^. All mice were housed in the specific pathogen-free animal facility at Wuhan University and all animal experiments were in accordance with protocols approved by the Institutional Animal Care and Use Committee of Wuhan University.

### Yeast two-hybrid assays

The pGBT9-MAVS vector and the individual pGADT7 vectors encoding E3s were transformed into AH109 competent cells which were grown on the Trp^−^Leu^−^ medium plates at 30 °C for 2 days. The positive clones were transferred to the Ade^−^His^−^Trp^−^Leu^−^ medium plates and cultured at 30 °C for 3–5 days. The positive clones were recognized as potential MAVS-interacting E3s.

### Reagents, antibodies, and constructs

Poly(I:C) and dsDNA were described previously^28^. H129-G4 was previously described and kindly provided by Dr. Min-Hua Luo (Wuhan Institute of Virology, Chinese Academy of Sciences)^65^. CHX and Baf-A1 were purchased from Sigma. MG132 was purchased from Topscience. Recombinant hM-CSF was purchased from Peprotech. The antibodies or control IgG were purchased from the indicated manufacturer’s, including mouse control IgG (Santa Cruz Biotechnology, sc-2025), rabbit control IgG (Millipore, 12-370), HRP-conjugated goat anti-mouse or rabbit IgG (Thermo Scientific, PA1-86717 and SA1-9510), HRP-conjugated mouse anti-FLAG (Sigma, A8592), HRP-conjugated goat anti-mouse IgG, F(ab′) 2 fragment specific (Jackson Immuno Research, 115-035-006), HRP-conjugated goat anti-rabbit IgG, F(ab′)2 fragment specific (Jackson Immuno Research, 111-035-006), mouse anti-FLAG (Sungene, KM8002), anti-GFP (Sungene, KM8009), anti-β-Actin (Sungene, KM9001), anti-GAPDH (Sungene, KM9002), anti-Tubulin (Sungene, KM9003), anti-HA (COVANCE, MMS-101R), anti-Ubiquitin (sc-8017), anti-p-IκBα (Cell Signaling Technologies, 9246L), anti-mouse MAVS (sc-365333), and anti-IRF3 (sc-33641); Rabbit anti-ubiquitin K48-specific linkage (Millipore, 05-1307), anti-ubiquitin K63-specific linkage (Millipore, 05-1308), anti-TBK1 (Abcam, 96328-11), anti-p-TBK1 (Abcam, 109272), anti-IRF3 (sc-9082), anti-p-IRF3 (Cell Signaling Technologies, 4947S), anti-p65 (sc-8008), anti-p-p65 (Cell Signaling Technologies, 3033S), anti-IκBα (sc-371), anti-STING (Cell Signaling Technologies, 13647S), anti-AIF (sc-13116), anti-Caspase 3 (Cell Signaling Technologies, 9662S), anti-Calreticulin (Abcam, ab2907), anti-RNF115 (Abcam, 187642), and anti-human MAVS (sc-166583). The cDNAs encoding human E3 ligases were amplified and cloned into the pGADT7 vector. The ISRE and IFN-β promoter luciferase reporter constructs, mammalian expression plasmids for MAVS, MAVS truncations, MITA, MITA truncations, TBK1, IRF3, RIG-I, ubiquitin, and ubiquitin mutants were previously described^28,35^. Mammalian expression plasmids for phage-6tag-RNF115, RNF115 mutants, or truncations, phage-6tag-ubiquitin or ubiquitin mutants, and FLAG-tagged MAVS, MAVS-TM(VAMP2), or MAVS-TM(CAAX) were constructed by standard molecular biology techniques.

### Generation and transfection of human CD14^+^ monocyte-derived macrophages

The protocol of human participation and blood donation was reviewed and approved by the Ethical Committee of Medical School of Wuhan University. Written, informed consent was obtained from all participants before the blood donation. Peripheral blood monocytes (PBMCs) were prepared from fresh blood of healthy donors with Ficoll-Plaque^TM^ PLUS following the manufacturer’s instructions (GE Healthcare, 17-1440-02). The CD14^+^ monocytes in PBMCs were isolated by a human CD14-positive selection kit (Stem Cell, 17858). About 37 × 10^6^ CD14^+^ monocytes were obtained from ~350 ml blood. These cells were cultured in RPMI1640 medium containing 10% FBS, 1% streptomycin–penicillin and rhM-CSF (20 ng/ml) immediately after isolation. The medium were changed on day 3. On day 5, the cells were transfected with control siRNA or siRNF115 by the Ultra Fection 2.0 reagent (4A Biotech, FXP092-010). Twelve hours later, the medium was changed with fresh RPMI1640 medium containing 10% FBS, 1% streptomycin–penicillin, and rhM-CSF (20 ng/ml) and cultured for 2 days. The cells were re-seeded into 24-well plates (for qRT-PCR or ELISA, 1–2 × 10^5^/well) or six-well plates (for immunoblot, 3 × 10^6^/well) for overnight culture. The cells were left uninfected or infected with VSV or HSV-1 of 6–12 h followed by qRT-PCR, ELISA, or immunoblot analysis. Control siRNA (NC): 5′-UUCUCCGAACGUGUCACGUTT-3′; siRNF115: 5′-GCCGUGGCUAGAACUGCAUTT-3′.

### Co-immunoprecipitation and immunoblot assays

These experiments were performed as previously described^37,66^. Cells were collected and lysed for 15 min with 800 μl lysis buffer (20 mM Tris–HCl, pH 7.4–7.5, 150 mM NaCl, 1 mM EDTA, 1% Nonidet P-40) containing inhibitors for protease and phosphatases (Biotool). Cell lysates (700 μl) were incubated with a control IgG or specific antibodies and protein G agarose for 2–4 h. The immunoprecipitates were washed for three times by 1 ml lysis buffer and subject to immunoblot analysis. The rest of lysates (100 μl) were subject to immunoblot analysis to detect the expression of target proteins.

### Protein purification and GST pull-down assay

The plasmids encoding GST and GST-RNF115 (1–200 aa) were transformed into BL21 competent cells which were induced with IPTG (1 mM) at 18 °C for 16 h. The cells were lysed in lysis buffer (20 mM Tris–HCl, 200 mM NaCl, 5% glycerol, and 0.3% Triton X-100) and the proteins were purified through affinity chromatography using a glutathione-Sepharose matrix (Transgen Biotech) followed by glutathione (10 mM in 50 mM Tris–HCl) elution and dialysis. FLAG-MAVS proteins were expressed with TNT Quick Coupled Transcription/Translation Systems kit (Promega, Madison, WI) as the manufacturer’s instructions. The purified GST or GST-RNF115 (1–200 aa) (5 μg) were incubated with FLAG-MAVS at 4 °C for overnight followed by glutathione agarose pull-down for 2 h in PBS containing protease inhibitors. The glutathione agarose was washed three times with PBS and subject to immunoblot analysis.

### Ubiquitination assays

Cells were lysed in regular lysis buffer (100–200 μl) and the cell lysates were denatured at 95 °C for 5 min in the presence of 1% SDS. A portion of cell lysates (20 μl) were saved for immunoblot analysis to detect the expression of target proteins. The rest of cell lysates (80–180 μl) were diluted with 1–2 ml lysis buffer and immunoprecipitated (Denature-IP) with either anti-FLAG beads or with protein G (20 μl) plus anti-FLAG or anti-MAVS or anti-MITA (0.2–0.5 μg). The immunoprecipitates were washed three times and subject to immunoblot analysis. For in vitro ubiquitination experiments, proteins were expressed with TNT Quick Coupled Transcription/Translation Systems kit (Promega, Madison, WI) as the manufacturer’s instructions. Ubiquitination was analyzed with an ubiquitination kit (Enzo Life Sciences, Farmingdale, NY) following the protocols recommended by the manufacturer.

### Cell fractionation assays

The cell fractionation assay was performed as previously described with a few changes^36^. The MLFs (6 × 10^7^) infected with VSV and HSV-1 or left uninfected were washed with PBS followed by dousing 20 times in 1 ml homogenization buffer (ApplyGen, Beijing, China) by 1 ml syringe. The homogenate was centrifuged at 500×*g* for 10 min. The supernatant (S5) was centrifuged at 5000×*g* for 10 min to precipitate mitochondria (P5K). The supernatant from this step (S5K) was further centrifuged at 50,000×*g* for 30 min to yield P50K, which contained the membrane fraction, and S50K, which was saved as cytosol.

### Transfection and reporter gene assays

HEK293 cells were transiently transfected with firefly luciferase reporter (100 ng) and TK-Renilla luciferase reporter (20 ng) and indicated plasmids or empty vector (100 ng) using standard calcium phosphate precipitation. After 24 h, luciferase assays were performed with a dual-specific luciferase reporter kit (Promega). The activity of firefly luciferase was normalized by that of Renilla luciferase to obtain relative luciferase activity.

### qRT-PCR and ELISA

Total RNA was extracted from cells using TRIzol (Life Technologies), and the first-strand cDNA was reverse-transcribed with All-in-One cDNA Synthesis SuperMix (Biotool). Gene expression was examined with a Bio-Rad CFX Connect system by a fast two-step amplification program with 2× SYBR Green Fast qRT-PCR Master Mix (Biotool). The value obtained for each gene was normalized to that of the gene encoding β-actin. The sequences of primers used in this study were included in Supplementary Table 1. The IFN-β, IL-6, TNF (Biolegend), and CCL5 (4A Biotech) protein in the sera or cell supernatants were determined by ELISA kits from the indicated manufacturers.

### Cell culture

Bone marrow cells were isolated from femurs of *Rnf115*^+/+^ and *Rnf115*^−/−^ mice. Primary MEFs were prepared from E14.5 embryos. The cells were cultured in DMEM containing 15% (vol/vol) FBS, 1% streptomycin–penicillin. GM-CSF (20 ng/ml, Peprotech) and M-CSF (10 ng/ml, Peprotech) were added to the bone marrow culture for differentiation of BMDCs and BMDMs. THP-1, HEK293, and HeLa cells were from the American Type Culture Collection, authenticated by STR locus analysis and tested for mycoplasma contamination^37^. The *Irf3*^−/−^*Irf*7^−/−^ and *p65*^−/−^ MEFs were kindly provided by Drs. Pinghui Feng (University of Southern California) and Tom Maniatis (Columbia University) as previously described^67^. Primary MLFs were isolated from ~8 to 10-week-old mice. Lungs were minced and digested in calcium and magnesium-free HBSS buffer supplemented with 10 mg/ml type I collagenase (Worthington) and 20 μg/ml DNase I (Sigma-Aldrich) for 3 h at 37 °C with shaking. Cell suspensions were cultured in DMEM containing 15% (vol/vol) FBS, 1% streptomycin–penicillin. Two days later, adherent fibroblasts were rinsed with PBS and cultured for experiments.

### Lentivirus-mediated gene transfer

HEK293 cells were transfected with phage-6tag-RNF115, phage-6tag-RNF115(2CA), phage-6tag-MAVS, phage-6tag-MAVS(K500R), phage-6tag-MITA, phage-6tag-MITA(3KR) or the empty vector along with the packaging vectors psPAX2 and pMD2G. The medium was changed with fresh full medium (15% FBS, 1% streptomycin–penicillin) at 8 h after transfection. Forty hours later, the supernatants were harvested to infect MLFs followed by various analyses.

### Viral infection

For qRT-PCR analysis, cells were seeded into 24-well plates (1–3 × 10^6^ cells per well) and infected with the specified viruses for the indicated time points. For viral replication assays, cells (1–3 × 10^6^) were infected with VSV-GFP or HSV-1-GFP. One hour later, the supernatants were removed and cells were washed twice with 1 ml pre-warmed PBS followed by culture in full medium for 12 h. Viral replication was analyzed by flow cytometry, fluorescent microscopy or qRT-PCR analysis. For the infection of mice, age-matched and sex-matched *Rnf115*^+/+^ and *Rnf115*^−/−^ mice were injected with EMCV (1.8 × 10^6^ PFU per mouse) or HSV-1 (1–3 × 10^7^ PFU per mouse) and the survival of animals was monitored every day. The hearts, spleens, or brains were collected for qRT-PCR analysis or plaque assays at 4 days after infection.

### Plaque assay

The supernatants of BMDCs or MLFs cultures and the homogenates of hearts, spleens, or brains from infected mice (or the serial dilutions) were used to infect monolayers of Vero cells. One hour later, the supernatants or homogenates were removed and the infected Vero cells were washed with pre-warmed PBS twice followed by incubation with DMEM containing 2% methylcellulose for 48 h. The cells were fixed with 4% paraformaldehyde for 15 min and stained with 1% crystal violet for 30 min before counting the plaques.

### shRNA

The shRNAs targeting RNF115 were constructed by plasmid pLentiLox 3.7 and transfected by Ultra Fection 2.0 (4A Biotech) or transferred by lentivirus into cells followed by qRT-PCR or immunoblot analysis. The shRNA sequences used in this study are as following: shRNF115#1: 5′-GCCATTTGGATCACACGATGT-3′; shRNF115#2: 5′-GGTTTAGAGTGTCCAGTATGC-3′

### Immunogold staining and electron microscopy

The experiments were performed as previously described with the help of Drs. Man-Li Wang and Zhi-Hong Hu (Wuhan Institute of Virology, Chinese Academy of Sciences)^68^. MLFs were fixed with 1% paraformaldehyde−0.5% glutaraldehyde for 10 min at 4 °C and re-fixed with 2% paraformaldehyde–2.5% glutaraldehyde for 1 h at 4 °C, washed three times with PBS and post-fixed in 1% osmium tetroxide in PBS buffer for 1 h at 4 °C. Cells were washed three times with PBS, dehydrated in graded ethanol and soaked in acetone. Infiltration was accomplished by using Spurr (Sigma) in gelatin capsules^69^. Ultrathin sections were immunostained with control IgG (1:50), anti-RNF115 (1:50), or anti-MAVS (1:50). Goat anti-rabbit IgG coated with gold particles (18 nm) and goat anti-mouse IgG coated with gold particles (12 nm) was used as the secondary antibody (1:50; Sigma). The samples were observed by use of a TEM (FEI Tecnai G2) operating at a 75-kV acceleration voltage.

### Immunofluorescence and confocal microscopy analysis

MLFs were cultured on coverslips and incubated with 250 nM Mito Tracker Red (Invitrogen) for 30 min at 37 °C. MLFs were fixed in 4% paraformaldehyde for 10 min and washed with PBS for three times. After that, cells were fixed with 0.1% Triton X-100 on ice for 5 min, washed in PBS and blocked in 1% BSA for 20 min. The coverslips were incubated with 1% BSA containing primary antibodies for 1 h followed by PBS wash for three times. The cells were further stained with Alexa Fluor 488- or 594- conjugated secondary antibodies. Images were acquired on an Olympus FV1000 fluorescence microscope.

### In situ PLA

Fixed and permeabilized cells were incubated overnight at 4 °C with the following pairs of primary antibodies. The cells were washed and allowed to react with a pair of proximity probes (Sigma). The remainder of the in situ PLA protocol was performed according to the manufacturer’s instructions. Cells were examined under an Olympus FV1000 fluorescence microscope, and the Image Pro Plus 6.0 was used for quantitative analyses.

### Semi-denaturing detergent agarose gel electrophoresis (SDD-AGE)

SDD-AGE was performed according to a published protocol with minor modifications^70^. Cells were collected and lysed for 10 min with 200 μl lysis buffer (20 mM Tris–HCl, pH 7.4–7.5, 150 mM NaCl, 1 mM EDTA, 1% Nonidet P-40) containing inhibitors for protease and phosphotases (Biotool). After centrifugation for 10 min at 14,000×*g*, supernatants were collected and added with 1× sample buffer (0.5× TBE, 10% glycerol, 2% SDS, and 0.0025% bromophenol blue) and loaded onto a vertical 1.5% agarose gel (Bio-Rad). After electrophoresis in the running buffer (1× TBE and 0.1% SDS) for 60 min with a constant voltage of 100 V at 4 °C, the proteins were subject to immunoblot analysis.

### Statistical analysis

Differences between experimental and control groups were determined by Student’s *t*-test (where two groups of data were compared) or by two-way ANOVA analysis (where more than two groups of data were compared). *P* values < 0.05 were considered statistically significant. For animal survival analysis, the Kaplan–Meier method was adopted to generate graphs, and the survival curves were analyzed with log-rank analysis.

### Reporting summary

Further information on research design is available in the Nature Research Reporting Summary linked to this article.

## Supplementary information

Supplementary Information

Reporting Summary

## Data Availability

All the other data supporting the findings of this study are available within the article and its supplementary information files and from the corresponding author upon reasonable request. A reporting summary for this article is available as a Supplementary Information file. Source data are provided with this paper.

## Source data

Source Data
