# Supplementary material for: RNF115 plays dual roles in innate antiviral responses by catalyzing distinct ubiquitination of MAVS and MITA
Source: Nat Commun. 2020 Nov 2;11:5536. doi: 10.1038/s41467-020-19318-3 (PMC7606512; doi:10.1038/s41467-020-19318-3)
Supplement: Supplementary file 1 — Supplementary Information [file 41467_2020_19318_MOESM1_ESM.pdf]

Supplementary Information

RNF115 plays dual roles in innate antiviral responses by catalyzing distinct ubiquitination of MAVS and MITA

Zhang et al

Supplementary Figure 1

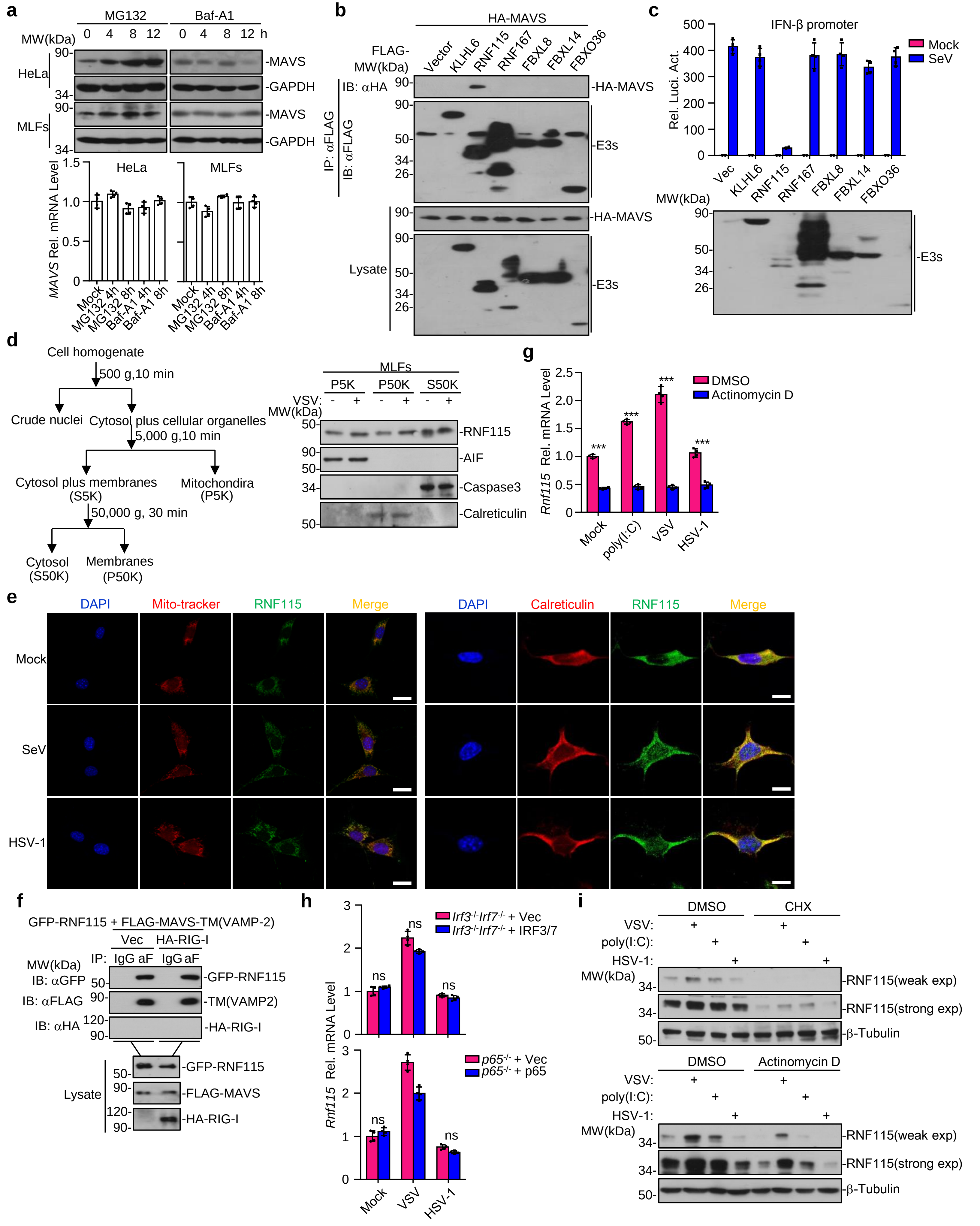

Supplementary Figure 1 RNF115 is localized on mitochondria and ER and interacts with MAVS.

(**a**) Immunoblot analysis (with anti-MAVS and GAPDH, upper panels) and qRT-PCR analysis of *MAVS* mRNA (lower graphs) in HeLa cells or in MLFs treated with MG132 for 0-12 h.

(**b**) Immunoprecipitation (with anti-FLAG) and immunoblot analysis (with anti-FLAG and HA) in HEK293 cells that were transfected with plasmids encoding HA-MAVS and FLAG-tagged E3s (KLHL6: 75 kD; RNF115: 37kD; RNF167: 45; FBXL8: 42; FBXL14: 42; FBXO36: 22 kD) for 24 h.

(**c**) Luciferase reporter assays analyzing IFN-β promoter activity (upper graph) and immunoblot analysis of FLAG-tagged E3s (lower panel) of HEK293 cells transfected with the indicated plasmids for 24 h followed by SeV infection for 8 h.

(**d**) Cell fractionation and immunoblot analysis of the subcellular fractions. Cell fractionation strategy is shown in the left. MLFs were left uninfected or infected with VSV for 4 h and cell fractions were analyzed by immunoblots with the indicated antibodies.

(**e**) Immunofluorescent staining of RNF115 (green) and mito-tracker (red) or Calreticulin (red) in MLFs left uninfected or infected with HSV-1 or SeV for 6 h.

(**f**) Immunoprecipitation (with anti-FLAG) and immunoblot analysis (with anti-FLAG, RNF115, or HA) in HEK293 cells that were transfected with plasmids encoding RNF115, FLAG-MAVS-TM(VAMP-2), and empty vector or HA-RIG-I for 24 h.

(**g**) qRT-PCR analysis of *Rnf115* mRNA in MEFs left untreated, transfected with poly(I:C), or infected with VSV or HSV-1 in the presence or absence of Act D (10 μg/ml) for 6 h.

(**h**) qRT-PCR of *Rnf115* mRNA in $Irf3^{-/-}Irf7^{-/-}$ MEFs stably transfected with an empty vector or IRF3 and IRF7 or in $p65^{-/-}$MEFs stably transfected with an empty vector or p65 followed by VSV or HSV-1 infection for 0-6 h.

(**i**) Immunoblot analysis (with anti-RNF115 and β-Tubulin) in MEFs transfected with poly(I:C) or infected with VSV or HSV-1 for 8 h in the presence or absence of CHX or Act D.

****P* <0.001 (two-tailed student's *t*-test). Scale bars represent 10 μm. Data are representative of three independent experiments (Graphs show mean ± S.D. in **a**, **c**, **g** and **h**).

Supplementary Figure 2

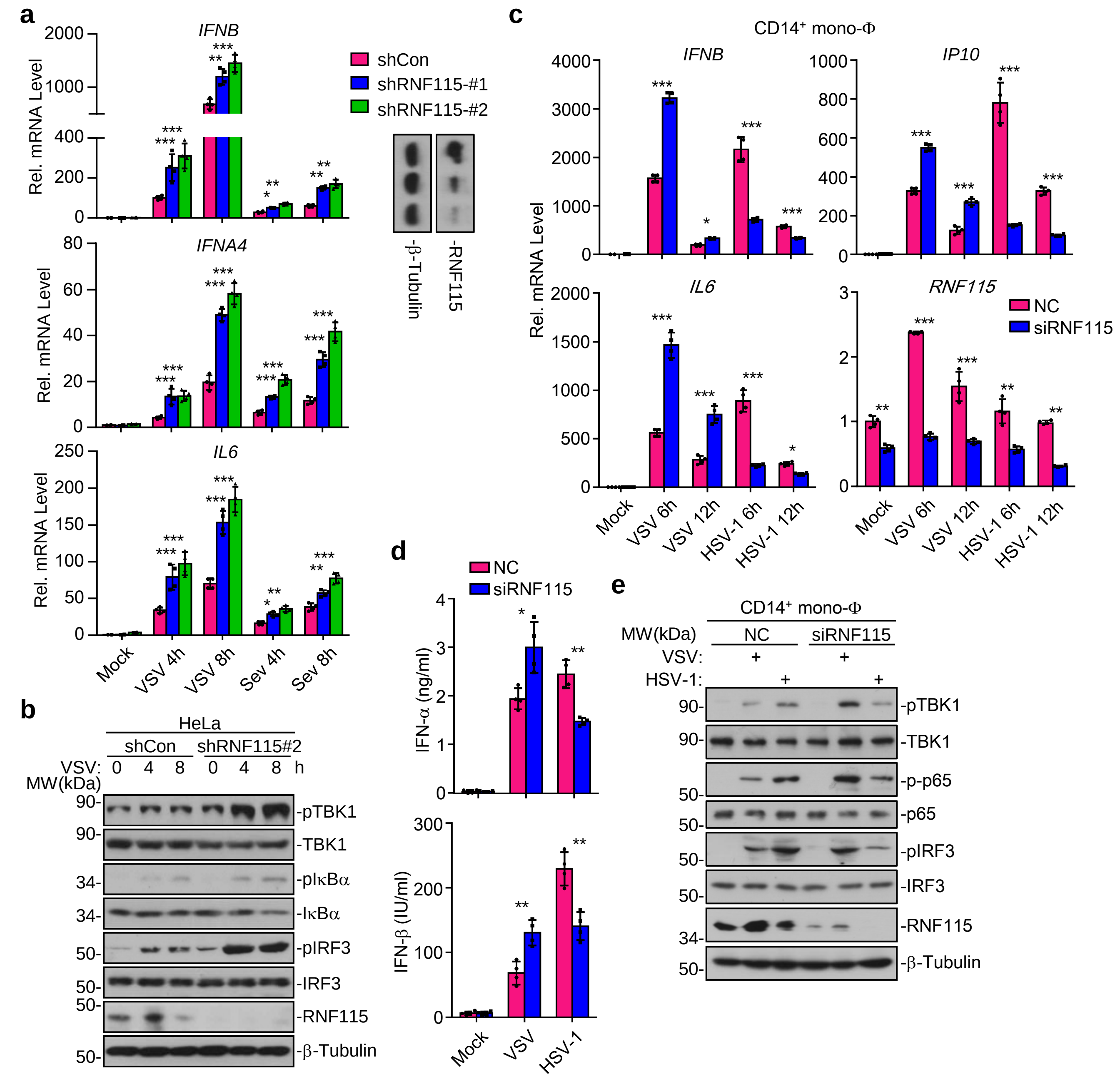

Supplementary Figure 2 Knockdown of RNF115 potentiates RNA virus-induced innate immune signaling.

(**a**) qRT-PCR analysis of *IFNB*, *IFNA4* or *IL6* mRNA in HeLa cells transfected with shCon, shRNF115#1 or shRNF115#2 followed by infection with SeV or VSV for 0-8 h (left graphs). The knockdown efficiency was examined by tmmunoblot analysis (with anti-RNF115 or anti-β-Tubulin) (right panels).

(**b**) Immunoblot analysis of total and phosphorylated (p-)IκBα, IRF3, TBK1, and RNF115 and β-Tubulin in HeLa cells transfected with shCon or shRNF115#2 for 36 h followed by infection with VSV for 0-8 h.

(**c**) qRT-PCR analysis of *IFNB*, *IP10*, *IL6* and *RNF115* mRNA in human primary $CD14^+$ monocytes-derived macrophages transfected with Control siRNA (NC) or siRNF115 for 72 h followed by VSV or HSV-1 infection for 0-12 h.

(**d**) ELISA analysis of IFN-α and IFN-β in human primary $CD14^+$ monocytes-derived macrophages transfected with Control siRNA (NC) or siRNF115 for 72 h followed by VSV or HSV-1 infection for 12 h.

(**e**) Immunoblot analysis of total and phosphorylated (p-)p65, IRF3, TBK1, and RNF115 and β-Tubulin in human primary $CD14^+$ monocytes-derived macrophages transfected with Control siRNA (NC) or siRNF115 for 72 h followed by VSV or HSV-1 infection for 0-6 h.

*P<0.05, **P < 0.01,***P <0.001 (two-way ANOVA or two-tailed student's *t*-test). Data are representative of three (a-b) or two (c-e) independent experiments (Graphs show mean ± S.D. in **a, c, d**).

Supplementary Figure 3

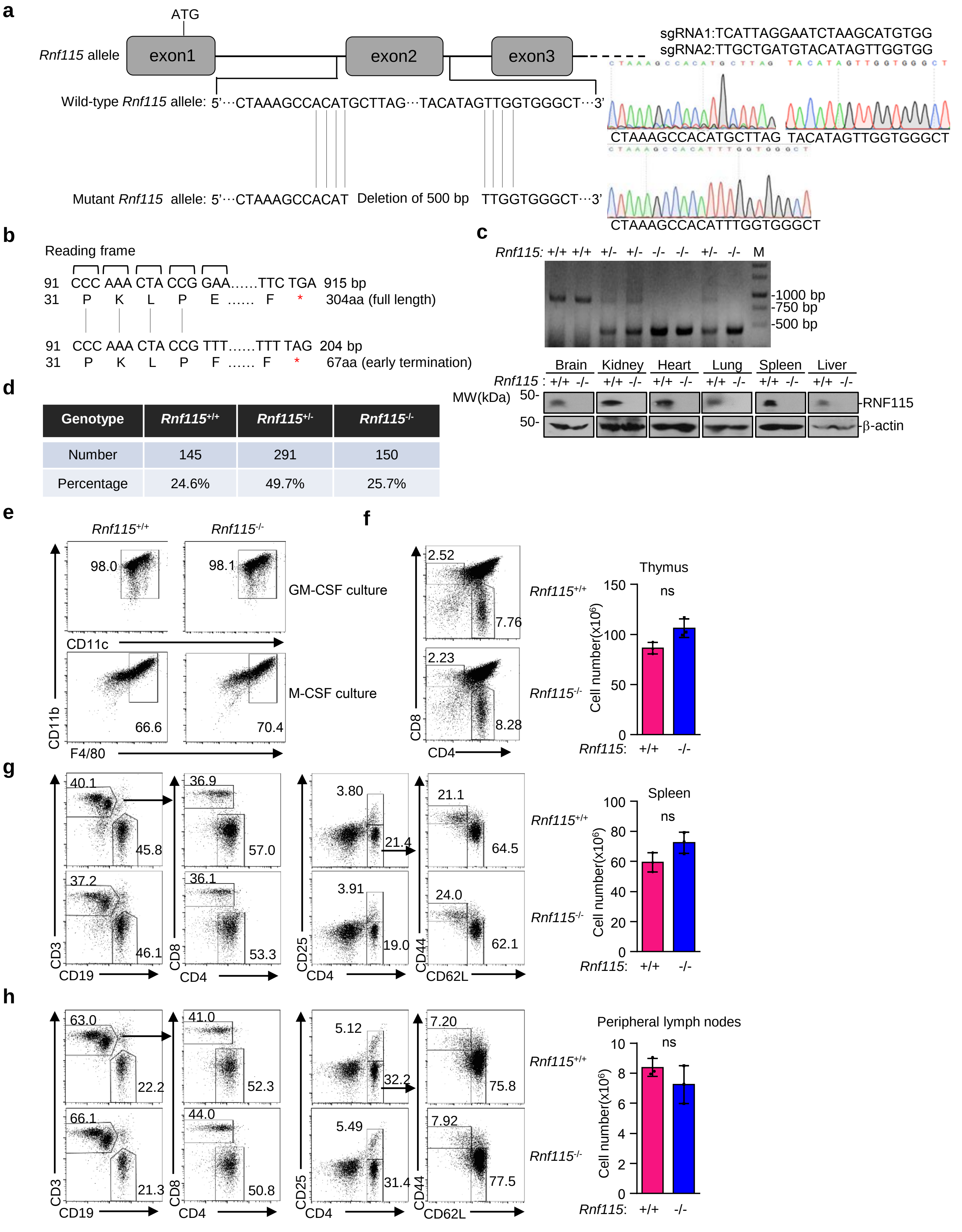

Genotype	*Rnf115*+/+	*Rnf115*+/-	*Rnf115*-/-
Number	145	291	150
Percentage	24.6%	49.7%	25.7%

Supplementary Figure 3 Generation of RNF115 KO mice.
(**a**) A scheme for CRIPSR/Cas9-mediated genome editing of the *Rnf115* gene locus.
(**b**) Gene sequence and reading frame of wild-type and edited *Rnf115* alleles.
(**c**) Genotyping analysis of *Rnf115*$^{+/+}$, *Rnf115*$^{+/-}$ and *Rnf115*$^{-/-}$ mice. Immunoblot analysis of RNF115 and β-Actin in brains, kidneys, hearts, lungs, spleens and livers from *Rnf115*$^{+/+}$ and *Rnf115*$^{-/-}$ mice.
(**d**) Mendelian inheritance statistics of the offspring of *Rnf115*$^{+/-}$ breeders.
(**e**) Flow cytometry analysis of in vitro generated *Rnf115*$^{+/+}$ and *Rnf115*$^{-/-}$ BMDCs or BMDMs .
(**f-h**) Flow cytometry analysis of immune cells and quantitative data in thymus (**f**), spleen (**g**) and peripheral lymph nodes (**h**) from *Rnf115*$^{+/+}$ and *Rnf115*$^{-/-}$ mice (n=3).
Data are representative of two independent experiments (Graphs show mean ± S.D. in **f -h**).

Supplementary Figure 4

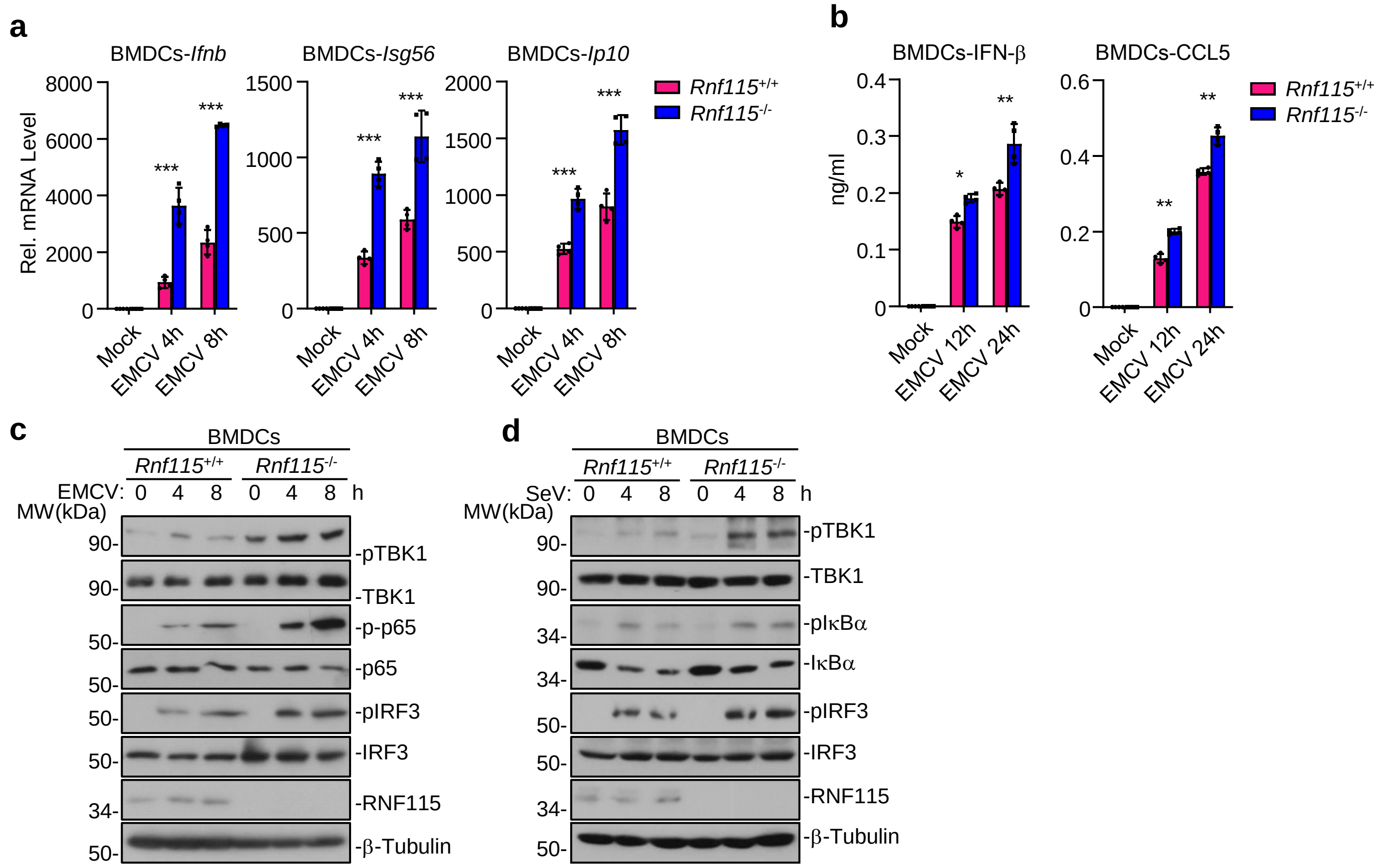

Supplementary Figure 4 Knockout of RNF115 potentiates EMCV-triggered innate immune signaling.
(**a**) qRT-PCR analysis of *Ifnb*, *Isg56*, or *Ip10* mRNA in *Rnf115*$^{+/+}$ and *Rnf115*$^{-/-}$ BMDCs infected with EMCV for 0–8 h.
(**b**) ELISA analysis of IFN-β and CCL5 in the supernatants of *Rnf115*$^{+/+}$ and *Rnf115*$^{-/-}$ BMDCs infected with EMCV for 12–24 h.
(**c**) Immunoblot analysis of total and phosphorylated (p-) p65, IRF3, and TBK1, RNF115 and β-Tubulin in *Rnf115*$^{+/+}$ and *Rnf115*$^{-/-}$ BMDCs infected with EMCV for 0-8 h.
(**d**) Immunoblot analysis of total and phosphorylated (p-) IκBα, IRF3, and TBK1, RNF115 and β-Tubulin in *Rnf115*$^{+/+}$ and *Rnf115*$^{-/-}$ BMDCs infected with SeV for 0-8 h.
P*<0.05, ***P* < 0.01,**P* <0.001 (two-tailed student's *t*-test). Data are representative of two independent experiments (Graphs show mean ± S.D. in **a** and **b**).

Supplementary Figure 5

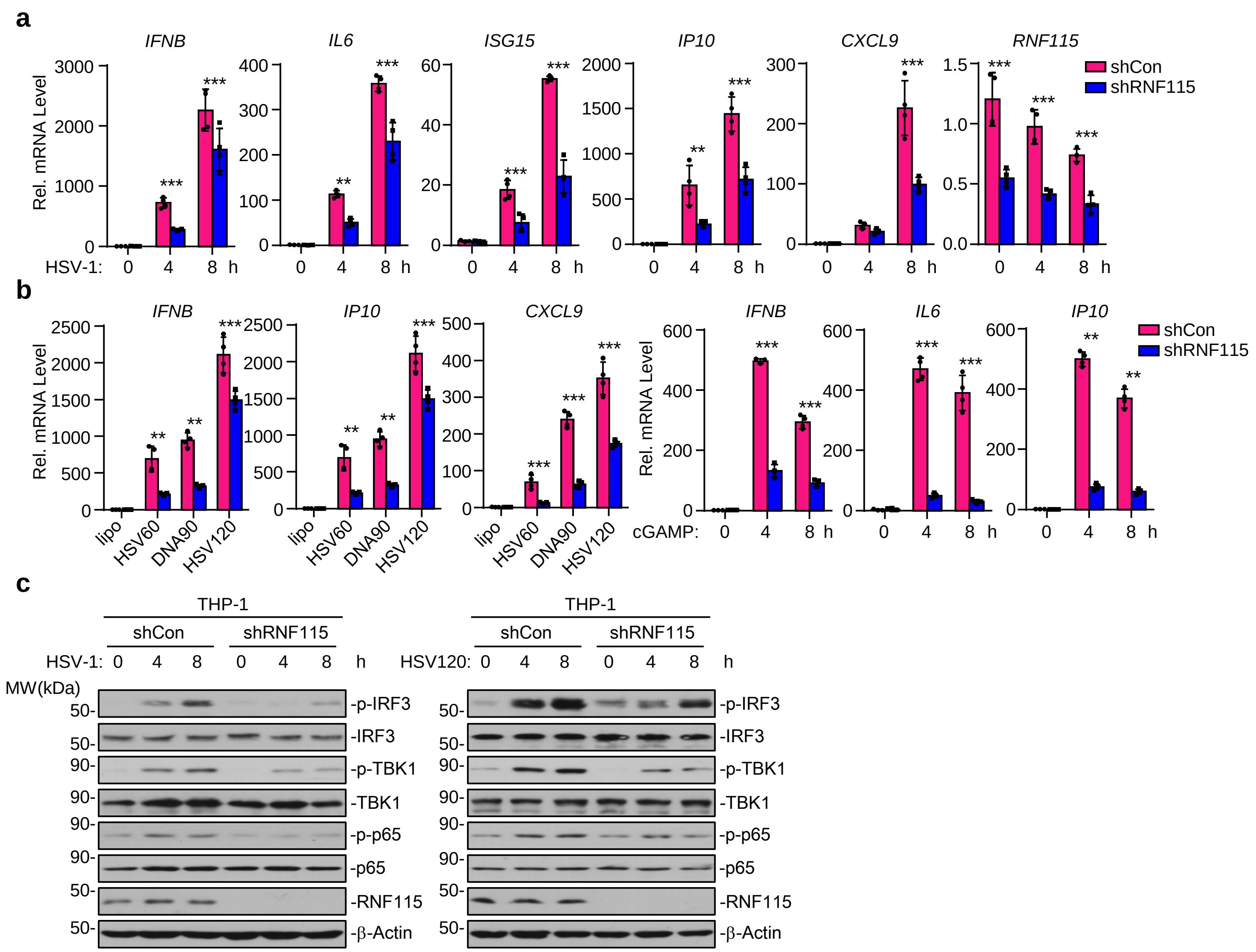

Supplementary Figure 5 Knockdown of RNF115 impairs HSV-1-triggered innate immune signaling.

(**a**) qRT-PCR analysis of *IFNB*, *IL6*, *ISG15*, *IP10*, *CXCL9* and *RNF115* mRNA in THP-1 cells stably transfected with shCon or shRNF115#2 followed by infection with HSV-1 for 0-8 h.

(**b**) qRT-PCR analysis of *IFNB*, *ISG56*, *CXCL9*, *IL6* and *ISG15* mRNA in THP-1 cells stably transfected with shCon or shRNF115#2 followed by transfection with HSV60, DNA90 and HSV120 (left graphs) or treated with digitonin-mediated cGAMP permeabilization for 0-8 h.

(**c**) Immunoblot analysis of total and phosphorylated (p-) IRF3,TBK1, p65, and RNF115 and β-Actin in THP-1 cells stably transfected with shCon or shRNF115#2 followed by infection with HSV-1 or transfected with HSV120 for 0-8 h.

*P < 0.05,**P < 0.01,***P <0.001 (two-tailed student's *t*-test). Data are representative of two independent experiments (Graphs show mean ± S.D. in **a** and **b**).

Supplementary Figure 6

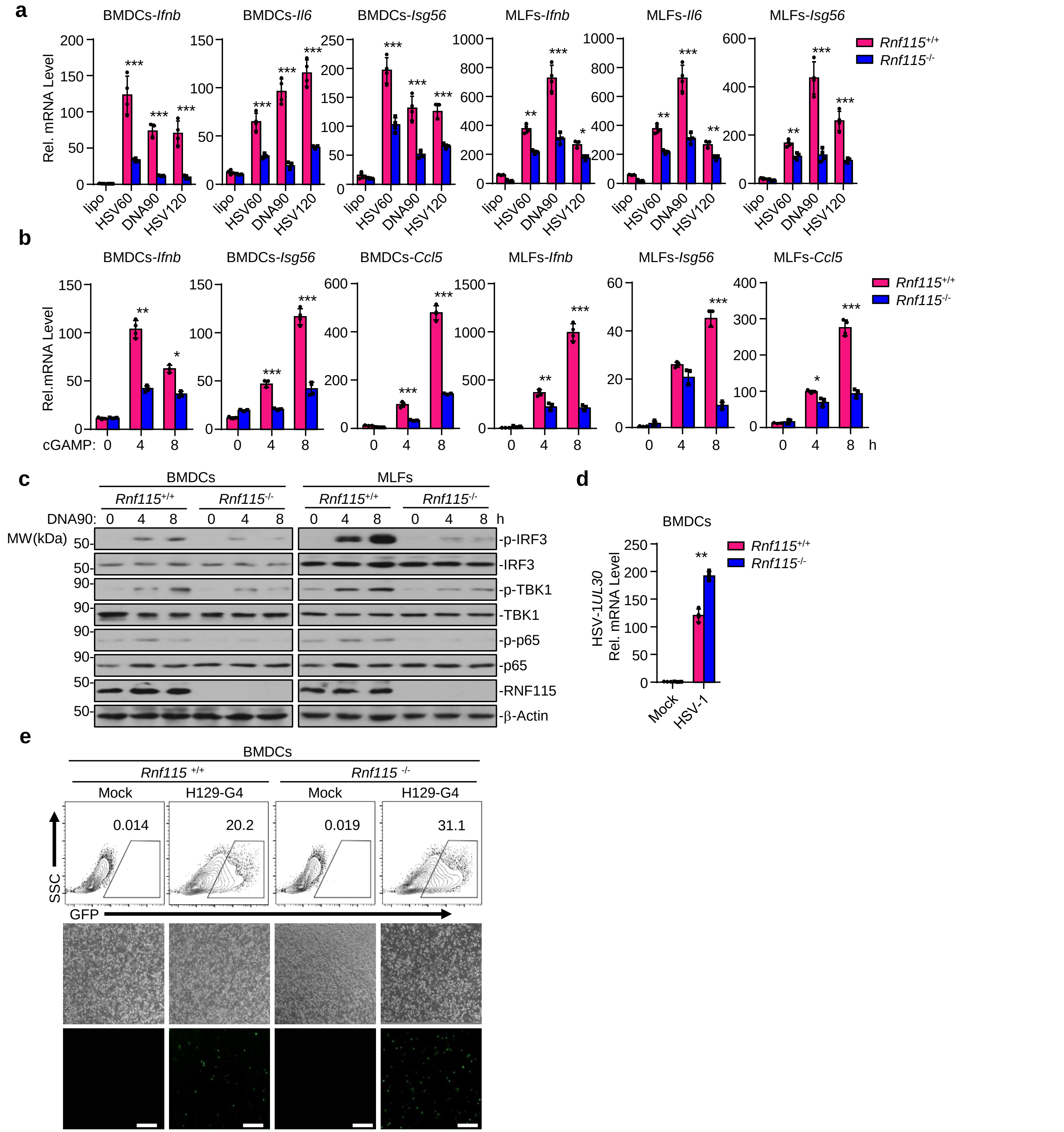

Supplementary Figure 6 Knockout of RNF115 impairs HSV-1-triggered innate immune signaling.

(**a**) qRT-PCR analysis of *Ifnb*, *Il6* and *Isg56* mRNA in *Rnf115*$^{+/+}$ and *Rnf115*$^{-/-}$ BMDCs or MLFs transfected with HSV60, DNA90 and HSV120 for 8 h.

(**b**) qRT-PCR analysis of *Ifnb*, *Isg56* and *Ccl5* mRNA in *Rnf115*$^{+/+}$ and *Rnf115*$^{-/-}$ BMDCs or MLFs treated with digitonin-mediated cGAMP permeabilization for 0-8 h.

(**c**) Immunoblot analysis of total and phosphorylated (p-) IRF3, TBK1, and p65, and RNF115 and β-Actin in *Rnf115*$^{+/+}$ and *Rnf115*$^{-/-}$ BMDCs or MLFs transfected with DNA90 for 0-8 h.

(**d**) qRT-PCR analysis of HSV-1 *UL30* mRNA in *Rnf115*$^{+/+}$ and *Rnf115*$^{-/-}$ BMDCs infected with HSV-1 (MOI = 0.5) for 1 hour followed by twice PBS wash and cultured in full medium for 24 hours.

(**e**) Flow cytometry analysis (upper flow charts) and fluorescent microscopy imaging (lower images) of the replication of H129-G4 in *Rnf115*$^{+/+}$ and *Rnf115*$^{-/-}$ BMDCs left uninfected or infected with H129-G4 (MOI = 0.5) for 1 h followed by twice PBS wash and cultured in full medium for 24 h. Numbers adjacent to the outlined areas indicate percentages of GFP^{+} BMDCs.

P* < 0.05, ***P* < 0.01, ****P* <0.001 (two-tailed student's *t*-test). Scale bars represent 200 μm. Data are representative of three(a-d)** or two**(e)** independent experiments (Graphs show mean ± S.D. in **a**, **b**, **d**).

Supplementary Figure 7

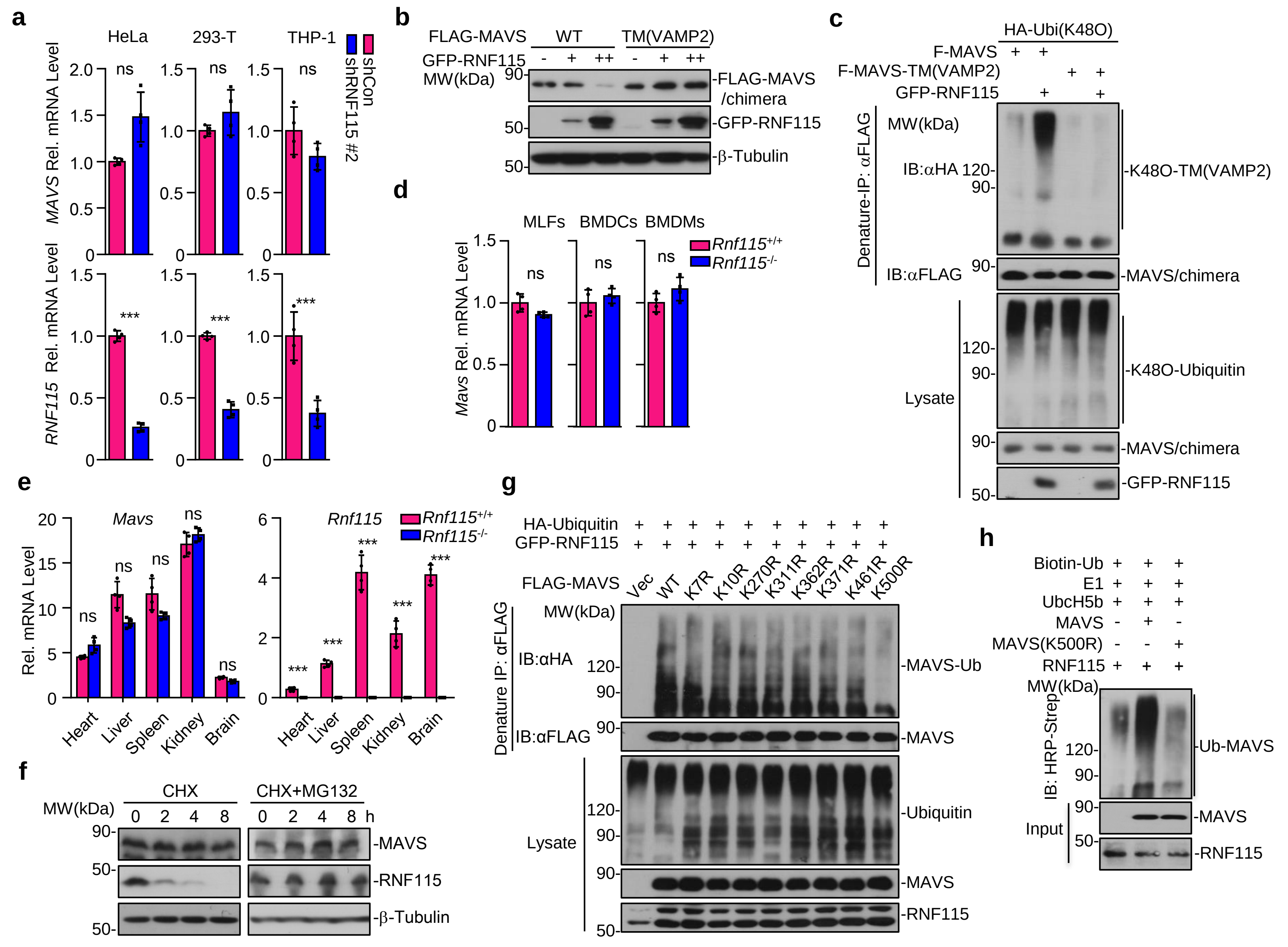

Supplementary Figure 7 RNF115 catalyzes K48-linked ubiquitination of MAVS at Lys500.

(**a**) qRT-PCR analysis of *Mavs* and *Rnf115* mRNA in HeLa cells transfected with shCon or shRNF115#2) for 36 h.

(**b**) Immunoblot analysis (with anti-FLAG, GFP or β-Tubulin) in HEK293 cells transfected with GFP-RNF115 and FLAG-MAVS or FLAG-MAVS-TM(VAMP2) for 24 h.

(**c**) Denature-IP (with anti-FLAG) and immunoblot analysis (with anti-FLAG, HA or GFP) in HEK293 cells transfected with plasmids encoding FLAG-MAVS-TM(VAMP2), HA-Ub-K48O and GFP-RNF115 for 24 h.

(**d**) qRT-PCR analysis of *Mavs* mRNA in *Rnf115*$^{+/+}$ and *Rnf115*$^{-/-}$ MLFs, BMDCs or BMDMs.

(**e**) qRT-PCR analysis of *Mavs* and *Rnf115* mRNA in hearts, livers, spleens, kidneys and brains from *Rnf115*$^{+/+}$ and *Rnf115*$^{-/-}$ mice.

(**f**) Immunoblot analysis of MAVS, RNF115, GAPDH or β-Tubulin in MLFs followed by CHX (upper graphs) or CHX+MG132 (lower graphs) treatment for 0-8 h.

(**g**) Denature-IP (with anti-FLAG) and immunoblot analysis (with anti-FLAG, HA or GFP) in HEK293 cells transfected with plasmids encoding FLAG-MAVS or mutations, HA-tagged ubiquitin and the empty vector or GFP-RNF115 for 24 h.

(**h**) In vitro ubiquitination analysis of MAVS. MAVS, MAVS(K500R) and RNF115 were translated in vitro. Biotin-Ub, E1 and UbcH5 were added for ubiquitination assays. Ubiquitin-conjugated MAVS was detected by immunoblot with HRP–streptavidin (upper panel). The proteins in the input were detected by immunoblots with the indicated antibodies (lower panels).

***, P<0.001 (two-tailed student's t-test). Data are representative of two independent experiments (Graphs show mean ± S.D. in **a**, **d** and **e**).

Supplementary Figure 8

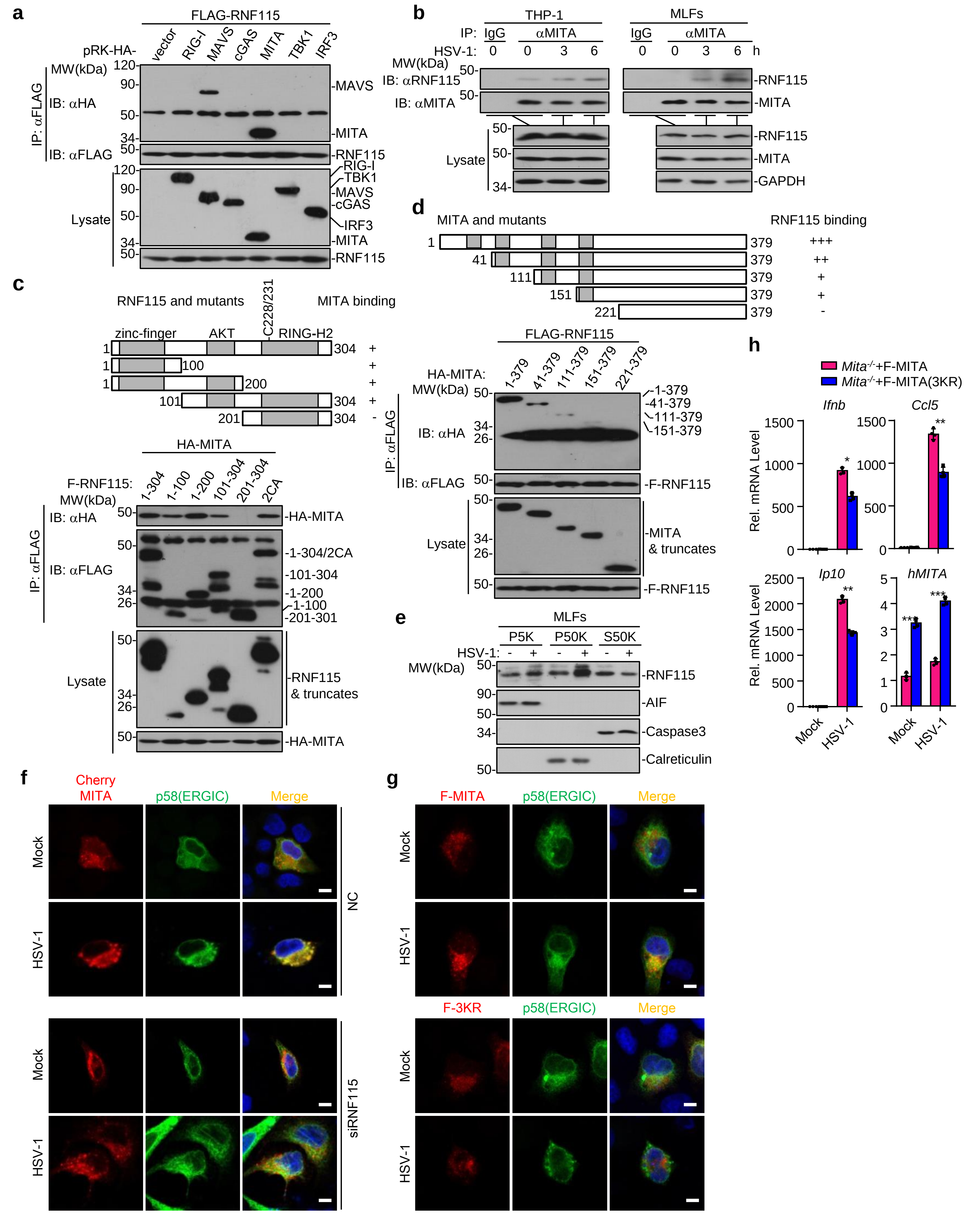

Supplementary Figure 8 RNF115 interacts with MITA after HSV-1 infection.
(**a**) Immunoprecipitation (with anti-FLAG) and immunoblot analysis (with anti-FLAG or HA) in HEK293 cells that were transfected with plasmids encoding FLAG-RNF115 and HA-tagged, RIG-I, MAVS, cGAS, MITA, TBK1 and IRF3 for 24 h.
(**b**) Immunoprecipitation (with anti-MITA) and immunoblot analysis (with anti-MITA, RNF115, or GAPDH) in THP-1 cells (left panels) or MLFs (right panels) that were left uninfected or infected with HSV-1 for 3-6 h.
(**c-d**) Immunoprecipitation (with anti-FLAG) and immunoblot analysis (with anti-FLAG or HA) in HEK293 cells that were transfected with plasmids encoding HA-MITA and FLAG-tagged RNF115 or truncates (**c**), or with plasmids encoding FLAG-tagged RNF115 and HA-MITA or mutants (**d**) for 24 h.
(**e**) Cell fractionation and immunoblot analysis of the subcellular fractions. MLFs were left uninfected or infected with HSV-1 for 4 hours and cell fractions were analyzed by immunoblots with the indicated antibodies.
(**f**) HeLa cells were transfected with the control siRNA or siRNF115 for 24 h followed by transfection of Cherry-MITA and p58-GFP (ERGIC marker) for 20 h. The cells were infected with HSV-1 for 0-6 h before fluorescent confocal microscopy analysis.
(**g**) HeLa cells were transfected with FLAG-tagged MITA or MITA(3KR) together with p58-GFP for 24 h followed by HSV-1 infection for 0-6 h before immunofluorescent analysis (with anti-FLAG) and fluorescent confocal microscopy analysis.
(**h**) qRT-PCR analysis of *Ifnb*, *Ccl5*, *Ip10* mRNA in *Mita*$^{-/-}$ MLFs reconstituted with MITA or MITA(3KR) followed by infection with HSV-1 for 0-6 h.
*$P < 0.05$, **$P<0.01$, ***$P<0.001$ (two tailed student's *t*-test). Scale bars represent 20 μm. Data are representative of three **(a, c, d, h)** or two**(b, f, g)** independent experiments (Graphs show mean ± S.D. in **h**).

Supplementary Table 1

Supplementary Table 1 Primers for qRT-PCR assays.

Gene	Forward	Reverse
GAPDH	GAGTCAACGGATTTGGTCGT	GACAAGCTTCCCGTTCTCAG
IFNB	TTGTTGAGAACCTCCTGGCT	TGACTATGGTCCAGGCACAG
IFNA4	GTTCCAGAAGGCTCAAGCCATC	TAGGAGGCTCTGTTCCCAAGCA
ISG15	AGGACAGGGTCCCCCTTGCC	CCTCCAGCCCGCTCACTTGC
IL6	AGACAGCCACTCACCTCTTCAG	TTCTGCCAGTGCCTCTTTGCTG
IP10	GGTGAGAAGAGATGTCTGAATCC	GTCCATCCTTGGAAGCACTGCA
CXCL9	CTGTTCCTGCATCAGCACCAAC	TGAACTCCATTCTTCAGTGTAGCA
RNF115	AGCTGACAAGGAAAAGATCACA	CTAGCCACGGCACAATACAAC
MAVS	ATGGTGCTCACCAAGGTGTCTG	TCTCAGAGCTGCTGTCTAGCCA
MITA	ATAAACTGCCCCAGCAGACC	CCCACAGTAACCTCTTCCTTTT
VSV-N	CCTTGCAGTGACATGACTGCTCTT	TGATAGTACCGGAGGATTGACGAC
UL30	CATCACCGACCCGGAGAGGGAC	GGGCCAGGCGCTTGTTGGTGTA
SeV-P	CAAAAGTGAGGGCGAAGGAGAA	CGCCCAGATCCTGAGATACAGA
β-Actin	ACGGCCAGGTCATCACTATT	TGGCATAGAGGTCTTTACGGA
Ifnb	TCCTGCTGTGCTTCTCCACCA	AAGTCCGCCCTGTAGGTGAGG
Isg15	GGCCACAGCAACATCTATGA	ACTGGGGCTTTAGGCCATAC
Isg56	GAAGTCCTTTGCTTGGAGGA	AGCCCAAGAAGGCTGGTACT
Ifna4	AGGATCACTGTGTACCTGAGA	TCTCCACACTTTGTCTCAGGA
Ip10	GTGAGAATGAGGGCCATAGG	TTTTTGGCTAAACGCTTTCAT
Il6	GGGAAATCGTGGAAATGAGAAA	ATCCAGTTTGGTAGCATCCATC
Ccl5	CTGCTGCTTTGCCTACCTCT	CTTGAACCCACTTCTTCTCTGG
Mavs	CTGCCAACACAATACCACCTGAG	TCTCTGGTCCAGAGTGCAAGCT
Rnf115	GTCCCAGATGTGACTCAGGCTT	GGATTGCTACTTAGAAATGGTC
