# Supplementary material for: RNF115 plays dual roles in innate antiviral responses by catalyzing distinct ubiquitination of MAVS and MITA
Source: Nat Commun. 2020 Nov 2;11:5536. doi: 10.1038/s41467-020-19318-3 (PMC7606512; doi:10.1038/s41467-020-19318-3)
Supplement: Supplementary file 2 — Reporting Summary [file 41467_2020_19318_MOESM2_ESM.pdf]

nature research

Corresponding author(s): Bo Zhong

Last updated by author(s): Aug 24, 2020

Reporting Summary

Nature Research wishes to improve the reproducibility of the work that we publish. This form provides structure for consistency and transparency in reporting. For further information on Nature Research policies, see Authors & Referees and the Editorial Policy Checklist .

Statistics

For all statistical analyses, confirm that the following items are present in the figure legend, table legend, main text, or Methods section.

n/a	Confirmed	
☐	☒	The exact sample size (*n*) for each experimental group/condition, given as a discrete number and unit of measurement
☐	☒	A statement on whether measurements were taken from distinct samples or whether the same sample was measured repeatedly
☐	☒	The statistical test(s) used AND whether they are one- or two-sided<br>*Only common tests should be described solely by name; describe more complex techniques in the Methods section.*
☒	☐	A description of all covariates tested
☒	☐	A description of any assumptions or corrections, such as tests of normality and adjustment for multiple comparisons
☐	☒	A full description of the statistical parameters including central tendency (e.g. means) or other basic estimates (e.g. regression coefficient) AND variation (e.g. standard deviation) or associated estimates of uncertainty (e.g. confidence intervals)
☐	☒	For null hypothesis testing, the test statistic (e.g. *F*, *t*, *r*) with confidence intervals, effect sizes, degrees of freedom and *P* value noted<br>*Give P values as exact values whenever suitable.*
☒	☐	For Bayesian analysis, information on the choice of priors and Markov chain Monte Carlo settings
☐	☒	For hierarchical and complex designs, identification of the appropriate level for tests and full reporting of outcomes
☒	☐	Estimates of effect sizes (e.g. Cohen's *d*, Pearson's *r*), indicating how they were calculated

Our web collection on statistics for biologists contains articles on many of the points above.

Software and code

Policy information about availability of computer code

Data collection	BD FACSDiVa Software v8.0.1.1 for FACS<br>Bio-Rad CFX Manager 3.1 for qRT-PCR.<br>Software ZEISS ZEN 3.1 installed on a LSM880 Carl Zeiss confocal microscopy for immunofluorescent and fluorescent imaging.
Data analysis	Prism 6 for graphs and statistical analysis.<br>Flowjo 10.6.2 for FACS plots.

For manuscripts utilizing custom algorithms or software that are central to the research but not yet described in published literature, software must be made available to editors/reviewers. We strongly encourage code deposition in a community repository (e.g. GitHub). See the Nature Research guidelines for submitting code & software for further information.

Data

Policy information about availability of data

All manuscripts must include a data availability statement. This statement should provide the following information, where applicable:

- Accession codes, unique identifiers, or web links for publicly available datasets
- A list of figures that have associated raw data
- A description of any restrictions on data availability

Source data are provided with this paper. The source data underlying Figs. 1b, c, e-i, 2a-c, e, 3a-c, 4b-l, 5a-c, e-h, j, 6, and 7 and Supplementary Figs. 1a-d, f-i, 2, 3c-h, 4, 5, 6a-d, 7 and 8a-e, h. are provided as a Source Data file. All the other data supporting the findings of this study are available within the article and its supplementary information files and from the corresponding author upon reasonable request.

Field-specific reporting

Please select the one below that is the best fit for your research. If you are not sure, read the appropriate sections before making your selection.

☒ Life sciences ☐ Behavioural & social sciences ☐ Ecological, evolutionary & environmental sciences

For a reference copy of the document with all sections, see nature.com/documents/nr-reporting-summary-flat.pdf

Life sciences study design

All studies must disclose on these points even when the disclosure is negative.

Sample size	The group sizes of the animals chosen are based on the numbers we used for previous publications, and the sizes are large enough to determine statistically significant effects.
Data exclusions	No data was excluded from our study.
Replication	The experiments were performed with 2-3 independent replications. The replication numbers were described in the corresponding figure legends. For each replication, age- and sex-matched mice were used.
Randomization	Age- and sex-matched mice were selected and included in different groups. Selection of mice was based on genotype without a plan for randomization.
Blinding	Data collection of most mouse experiments were performed in a double blinding manner. Cellular and biochemical experiments were not performed in a blinding manner.

Reporting for specific materials, systems and methods

We require information from authors about some types of materials, experimental systems and methods used in many studies. Here, indicate whether each material, system or method listed is relevant to your study. If you are not sure if a list item applies to your research, read the appropriate section before selecting a response.

Materials & experimental systems

n/a	Involved in the study	
☐	☒	Antibodies
☐	☒	Eukaryotic cell lines
☒	☐	Palaeontology
☐	☒	Animals and other organisms
☐	☒	Human research participants
☒	☐	Clinical data

Methods

n/a	Involved in the study	
☒	☐	ChIP-seq
☐	☒	Flow cytometry
☒	☐	MRI-based neuroimaging

Antibodies

Antibodies used	mouse control IgG (Santa Cruz Biotechnology, sc-2025), rabbit control IgG (Millipore, 12–370), HRP- conjugated goat-anti mouse or rabbit IgG (Thermo Scientific, PA1-86717 and SA1-9510), HRP-conjugated mouse anti-FLAG (Sigma, A8592), HRP-conjugated goat anti-mouse IgG, F(ab') 2 fragment specific (Jackson Immuno Research, 115-035-006), HRP conjugated goat anti-rabbit IgG, F (ab')2 fragment specific (Jackson Immuno Research, 111-035-006), mouse anti-FLAG (Sungene, KM8002), anti-GFP (Sungene, KM8009), anti--Actin (Sungene, KM9001), anti-GAPDH (Sungene, KM9002), anti-Tubulin (Sungene, KM9003), anti-HA (COVANCE, MMS-101R), anti-Ubiquitin (sc-8017), anti-p-IκB (Cell Singling Technologies, 9246L), anti-mouse MAVS (sc-365333) and anti-IRF3 (sc-33641), anti-ubiquitin K48-specific linkage (Millipore, 05-1307), anti-ubiquitin K63-specific linkage (Millipore, 05-1308), anti-TBK1 (Abcam, 96328-11), anti-p-TBK1 (Abcam, 109272), anti-IRF3 (sc-9082), anti-p-IRF3 (Cell Singling Technologies, 4947S), anti-p65 (sc-8008), anti-p-p65 (Cell Singling Technologies, 3033S), anti-IκB (sc-371), anti-STING (Cell Singling Technologies, 13647S), anti-AIF (sc-13116), anti-Caspase 3 (Cell Singling Technologies,9662S), anti- Calreticulin (Abcam, ab2907), anti-RNF115 (Abcam, 187642) and anti-human MAVS (sc-166583).
Validation	anti-RNF115, 187642, mouse/rat/human, WB, https://www.abcam.com/rnf115-antibody-epr14539-ab187642.html<br>Other antibodies were used and validated in our previously publications (Liuyu T, et al., Cell Res, 2019, 29(1):67-79; Cai Z et al., J Exp. Med., 2020, 217, 5: e20191174; Zhang Q et al., Cell Res, 2020, May 26, published online; Wang XM et al., Nat Cancer, 2020, July 6, published online.).

Eukaryotic cell lines

Policy information about cell lines

Cell line source(s)	THP-1, HEK293 and HeLa cells were from the American Type Culture Collection (Sun H et al., Nat Commun, 2017, 8: 15534).
Authentication	These cells were authenticated by STR locus analysis.
Mycoplasma contamination	The cells were tested Mycoplsma negative.
Commonly misidentified lines (See ICLAC register)	n/a.

Animals and other organisms

Policy information about studies involving animals; ARRIVE guidelines recommended for reporting animal research

Laboratory animals	The Rnf115-/- mice were generated by the CRISPR/Cas9-mediated genome editing (GemPharmatech Co. Ltd). In brief, the vectors encoding Cas9 (44758, Addgene) and guide RNAs (5'-TCATTAGGAATCTAAGCATGTGG-3'; 5'-TTGCTGATGTACATAGTTGGTGG-3') were in vitro transcribed into mRNA and gRNA followed by injection into the fertilized eggs that were transplanted into pseudopregnant mice. The targeted genome of F0 mice was amplified with PCR and sequenced and the chimeras were crossed with wild-type C57BL/6 mice to obtain the Rnf115+/- mice. The F1 Rnf115+/- mice were further crossed with wild-type C57BL/6 mice for at least three generations. Mice were genotyped by PCR analysis followed by sequencing and the resulted Rnf115+/- mice were crossed to generate Rnf115+/+ and Rnf115-/- mice. Age- and sex-matched Rnf115+/+ and Rnf115-/- littermates were blindingly randomized into groups for animal studies. The Mavs-/- and Mita-/- mice were previously described and kindly provided by Dr. Hong-Bing Shu (Wuhan University) (Xu L et al., J Immunol, 2012, 188(1): 248-258; Li Y et al., PNAS, 2012, 109(29): 11770-11775.).
Wild animals	This study did not involve wild animals.
Field-collected samples	This study did not involve samples collected from the field.
Ethics oversight	All animal experiments were in accordance with protocols approved by the Institutional Animal Care and Use Committee of Wuhan University.

Note that full information on the approval of the study protocol must also be provided in the manuscript.

Human research participants

Policy information about studies involving human research participants

Population characteristics	Two adults participated in blood donation in this study. Participate #1: Male, 37 years old, body temperature: 36.5oC, donated 180 ml blood with an empty stomach. Participant #2: Male, 25 years old, body temperature: 36.6oC, donated 190 ml blood with an empty stomach.
Recruitment	Two healthy male adults were recruited for blood donation. These two men had not been sick (such as fever, cold, or diarrhea) or hospitalization within two months before the day of blood donation. Written, informed content was obtained from the two participants before blood donation.
Ethics oversight	The Ethical Committee of Medical School of Wuhan University

Note that full information on the approval of the study protocol must also be provided in the manuscript.

Flow Cytometry

Plots

Confirm that:

- [x] The axis labels state the marker and fluorochrome used (e.g. CD4-FITC).
- [x] The axis scales are clearly visible. Include numbers along axes only for bottom left plot of group (a 'group' is an analysis of identical markers).
- [x] All plots are contour plots with outliers or pseudocolor plots.
- [x] A numerical value for number of cells or percentage (with statistics) is provided.

Methodology

Sample preparation	Single cell suspensions were incubated with fluorochrome-conjugated antibodies against surface markers in PBS containing 1.5% FBS for 20 min at 4oC and then washed. LIVE/DEAD Fixable Blue Dead Cell Stain Kit from Biolegend was used to exclude dead cells. Cells were then fixed for 30 min at 4oC using Biolegend Cytofix/Cytoperm and washed twice followed by flow cytometry analysis.The detailed experimental procedures were described in the methods session.
Instrument	BD Fortessa and Celesta
Software	Windows Flowjo 10.6.2 for data analysis; BD FACSDiVa v8.0.1.1 for cell collection
Cell population abundance	n/a
Gating strategy	Forward versus side scatter (FSC vs SSC) gating was used to identify cells of interest and exclude debris and dead cells. also, LIVE/DEAD Fixable Dead Cell Stain Kit from Biolegend was used to exclude dead cells. A forward scatter height (FSC-H) vs. forward scatter area (FSC-A) density plot was used to exclude doublets. For cytokine measurement, we used non-restimulated samples as negative controls.

☐ Tick this box to confirm that a figure exemplifying the gating strategy is provided in the Supplementary Information.
