# Supplementary material for: RNF115 plays dual roles in innate antiviral responses by catalyzing distinct ubiquitination of MAVS and MITA
Source: Nat Commun. 2020 Nov 2;11:5536. doi: 10.1038/s41467-020-19318-3 (PMC7606512; doi:10.1038/s41467-020-19318-3)
Supplement: Supplementary file 3 — Source Data [file 41467_2020_19318_MOESM3_ESM.zip › Source Data_Orignal uncropped gels.pptx]

## Slide 1
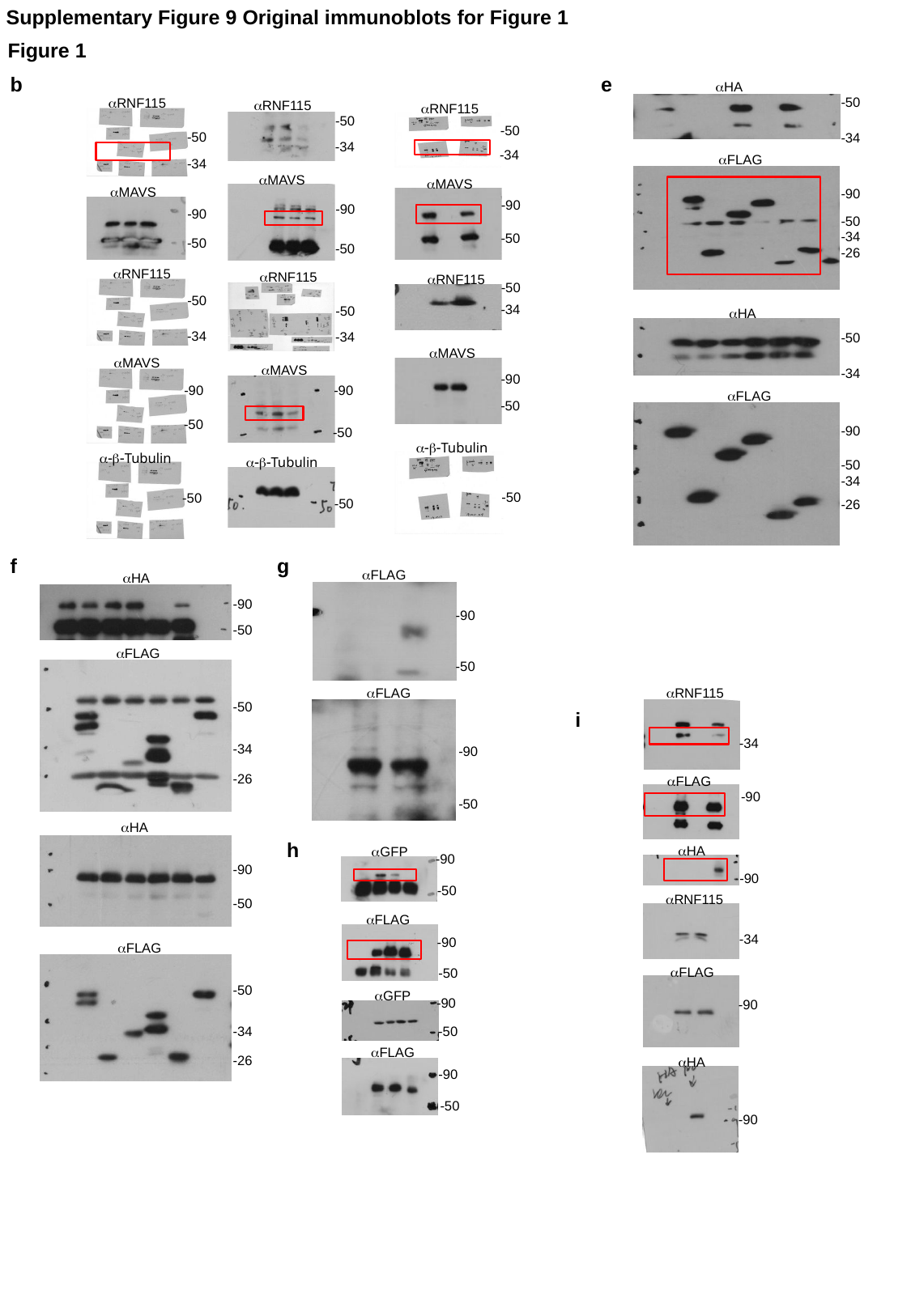

Supplementary Figure 9 Original immunoblots for Figure 1
 Figure 1
b
e
aHA
-50
aRNF115
aRNF115
aRNF115
-50
-50
-50
-34
-34
-34
aFLAG
-34
aMAVS
aMAVS
aMAVS
-90
-90
-90
-90
-50
-34
-50
-50
-50
-26
aRNF115
aRNF115
aRNF115
-50
-50
-34
-50
aHA
-34
-34
-50
aMAVS
aMAVS
aMAVS
-34
-90
-90
-90
aFLAG
-50
-50
-90
-50
a-b-Tubulin
a-b-Tubulin
a-b-Tubulin
-50
-34
-50
-50
-50
-26
f
g
aFLAG
aHA
-90
-90
-50
aFLAG
-50
aFLAG
aRNF115
-50
i
-34
-34
-90
-26
aFLAG
-90
-50
aHA
h
aHA
aGFP
-90
-90
-90
-50
aRNF115
-50
aFLAG
-34
-90
aFLAG
aFLAG
-50
-50
aGFP
-90
-90
-34
-50
aFLAG
-26
aHA
-90
-50
-90

## Slide 2
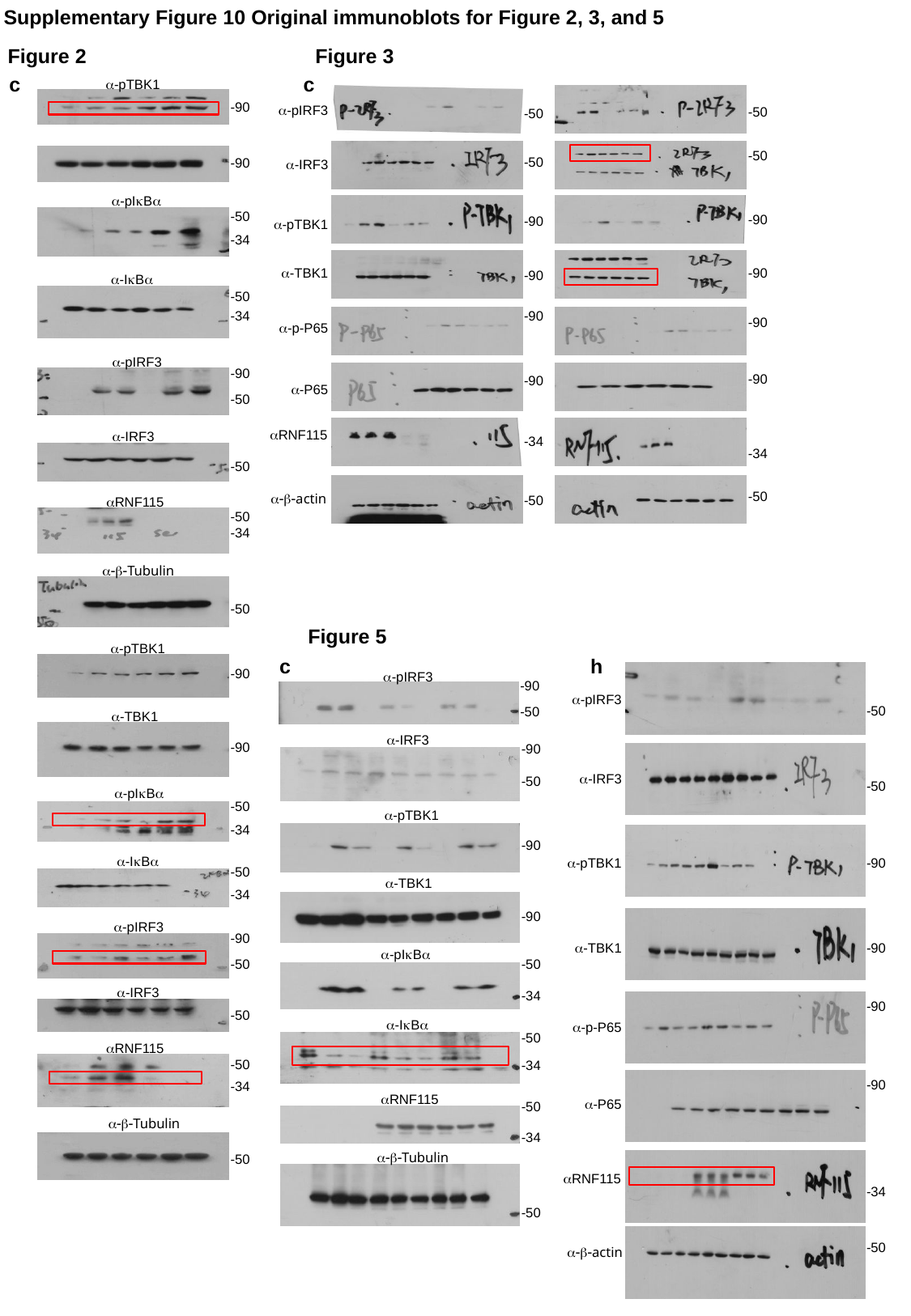

Supplementary Figure 10 Original immunoblots for Figure 2, 3, and 5
 Figure 2
 Figure 3
c
c
a-pTBK1
-90
a-pIRF3
-50
-50
-50
-50
-90
a-IRF3
a-pIkBa
-50
-90
-90
a-pTBK1
-34
-90
a-TBK1
-90
a-IkBa
-50
-90
-34
-90
a-p-P65
a-pIRF3
-90
-90
-90
a-P65
-50
aRNF115
a-IRF3
-34
-34
-50
a-b-actin
-50
-50
aRNF115
-50
-34
a-b-Tubulin
-50
 Figure 5
a-pTBK1
c
h
-90
a-pIRF3
-90
a-pIRF3
-50
-50
a-TBK1
a-IRF3
-90
-90
a-IRF3
-50
-50
a-pIkBa
-50
a-pTBK1
-34
-90
a-IkBa
a-pTBK1
-90
-50
a-TBK1
-34
-90
a-pIRF3
-90
a-TBK1
-90
a-pIkBa
-50
-50
a-IRF3
-34
-90
-50
a-IkBa
a-p-P65
-50
aRNF115
-50
-34
-90
-34
aRNF115
a-P65
-50
a-b-Tubulin
-34
a-b-Tubulin
-50
aRNF115
-34
-50
a-b-actin
-50

## Slide 3
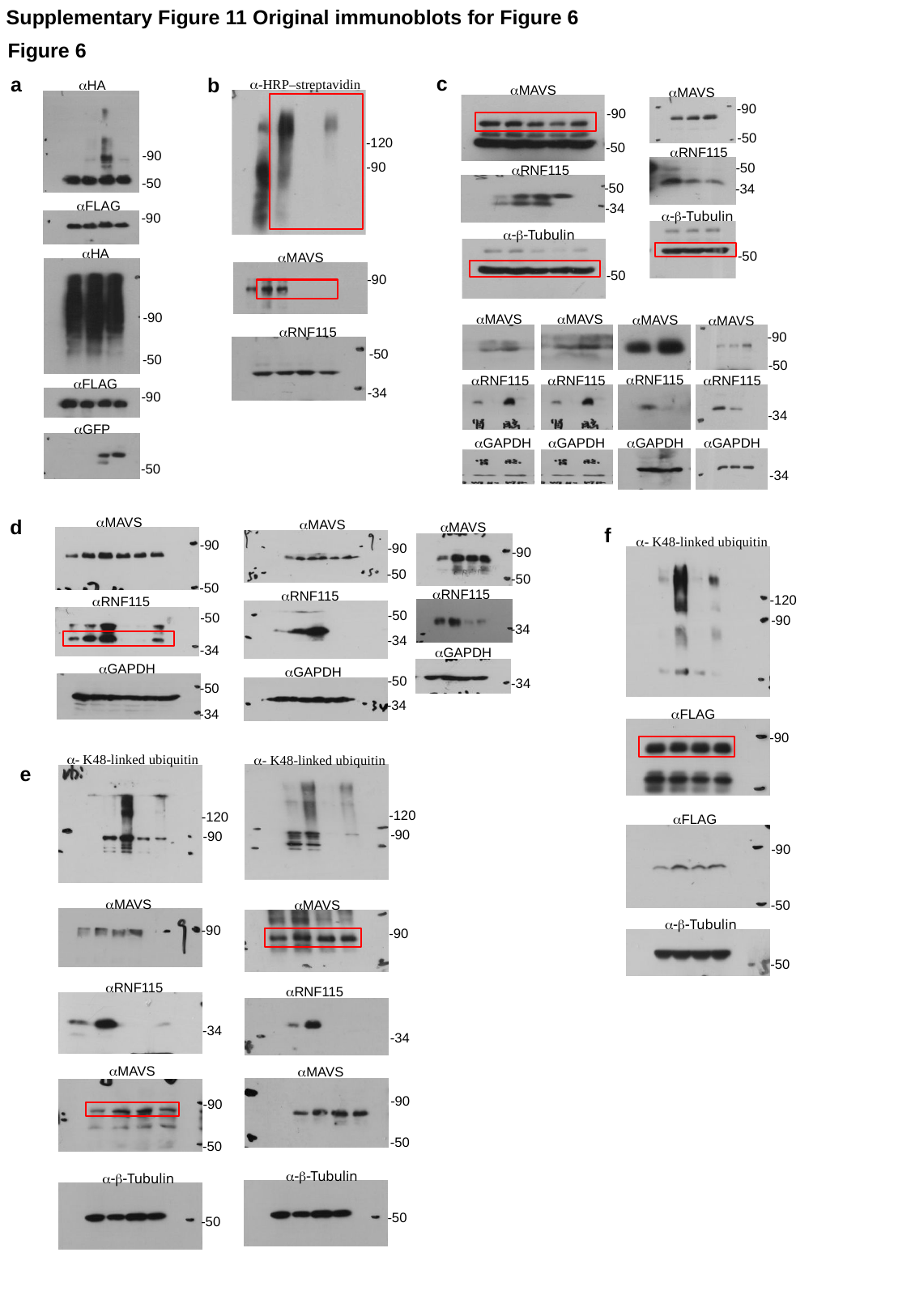

Supplementary Figure 11 Original immunoblots for Figure 6
 Figure 6
a-HRP–streptavidin
c
a
b
aHA
aMAVS
aMAVS
-90
-90
-50
-120
-50
aRNF115
-90
-90
-50
aRNF115
-50
-50
-34
aFLAG
-34
a-b-Tubulin
-90
a-b-Tubulin
aHA
-50
aMAVS
-50
-90
-90
aMAVS
aMAVS
aMAVS
aMAVS
aRNF115
-90
-50
-50
-50
aRNF115
aRNF115
aRNF115
aRNF115
aFLAG
-34
-90
-34
aGFP
aGAPDH
aGAPDH
aGAPDH
aGAPDH
-50
-34
aMAVS
d
aMAVS
aMAVS
f
a- K48-linked ubiquitin
-90
-90
-90
-50
-50
-50
aRNF115
aRNF115
-120
aRNF115
-50
-50
-90
-34
-34
-34
aGAPDH
aGAPDH
aGAPDH
-50
-34
-50
-34
-34
aFLAG
-90
a- K48-linked ubiquitin
a- K48-linked ubiquitin
e
-120
-120
aFLAG
-90
-90
-90
aMAVS
aMAVS
-50
a-b-Tubulin
-90
-90
-50
aRNF115
aRNF115
-34
-34
aMAVS
aMAVS
-90
-90
-50
-50
a-b-Tubulin
a-b-Tubulin
-50
-50

## Slide 4
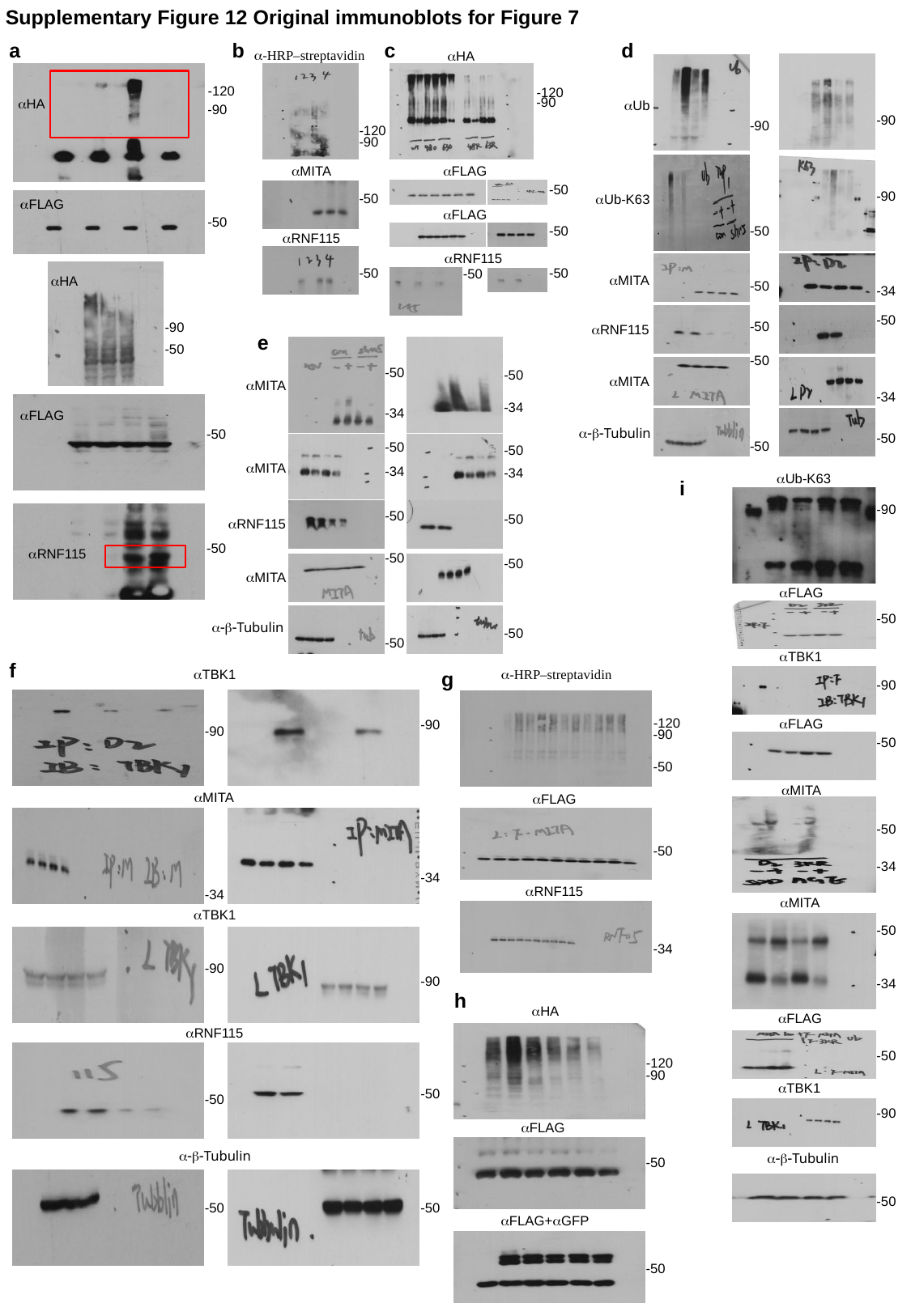

Supplementary Figure 12 Original immunoblots for Figure 7
a
b
c
d
a-HRP–streptavidin
aHA
-120
-120
-90
aHA
aUb
-90
-90
-90
-120
-90
aMITA
aFLAG
-50
-90
-50
aUb-K63
aFLAG
aFLAG
-50
-50
-50
aRNF115
aRNF115
-50
-50
-50
aMITA
aHA
-50
-34
-50
-50
-90
aRNF115
e
-50
-50
-50
-50
aMITA
aMITA
-34
-34
-34
aFLAG
a-b-Tubulin
-50
-50
-50
-50
-50
aMITA
-34
-34
aUb-K63
i
-90
-50
-50
aRNF115
-50
aRNF115
-50
-50
aMITA
aFLAG
-50
a-b-Tubulin
-50
-50
aTBK1
f
a-HRP–streptavidin
aTBK1
g
-90
-120
aFLAG
-90
-90
-90
-50
-50
aMITA
aMITA
aFLAG
-50
-50
-34
-34
aRNF115
-34
aMITA
aTBK1
-50
-34
-90
-90
-34
h
aHA
aFLAG
aRNF115
-50
-120
-90
aTBK1
-50
-50
-90
aFLAG
a-b-Tubulin
a-b-Tubulin
-50
-50
-50
-50
aFLAG+aGFP
-50

## Slide 5
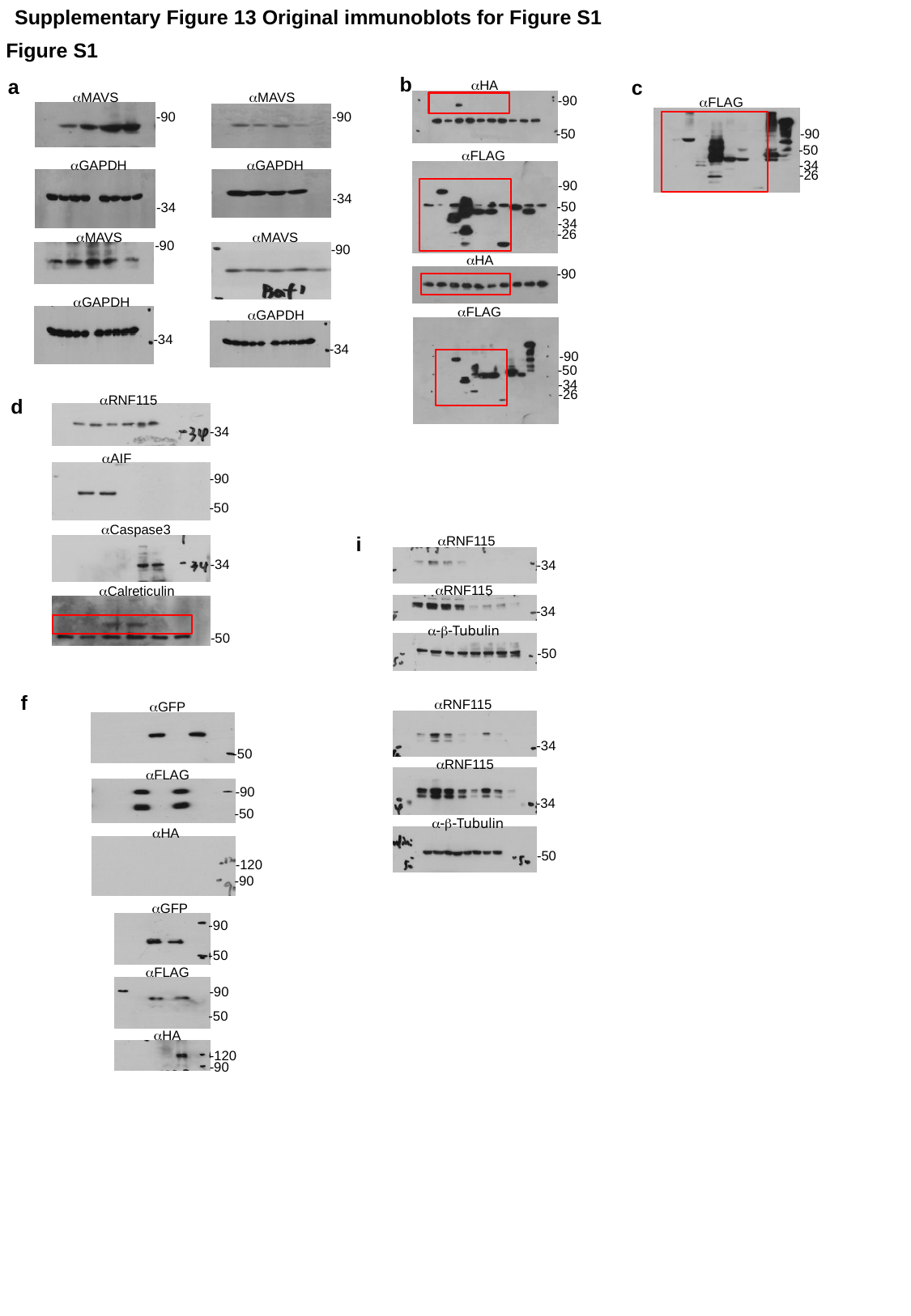

Supplementary Figure 13 Original immunoblots for Figure S1
 Figure S1
b
a
c
aHA
aMAVS
aMAVS
-90
aFLAG
-90
-90
-50
-90
-50
aFLAG
aGAPDH
aGAPDH
-34
-26
-90
-34
-50
-34
-34
-26
aMAVS
aMAVS
-90
-90
aHA
-90
aGAPDH
aFLAG
aGAPDH
-34
-34
-90
-50
-34
-26
aRNF115
d
-34
aAIF
-90
-50
aCaspase3
i
aRNF115
-34
-34
aRNF115
aCalreticulin
-34
a-b-Tubulin
-50
-50
f
aRNF115
aGFP
-34
-50
aRNF115
aFLAG
-90
-34
-50
a-b-Tubulin
aHA
-50
-120
-90
aGFP
-90
-50
aFLAG
-90
-50
aHA
-120
-90

## Slide 6
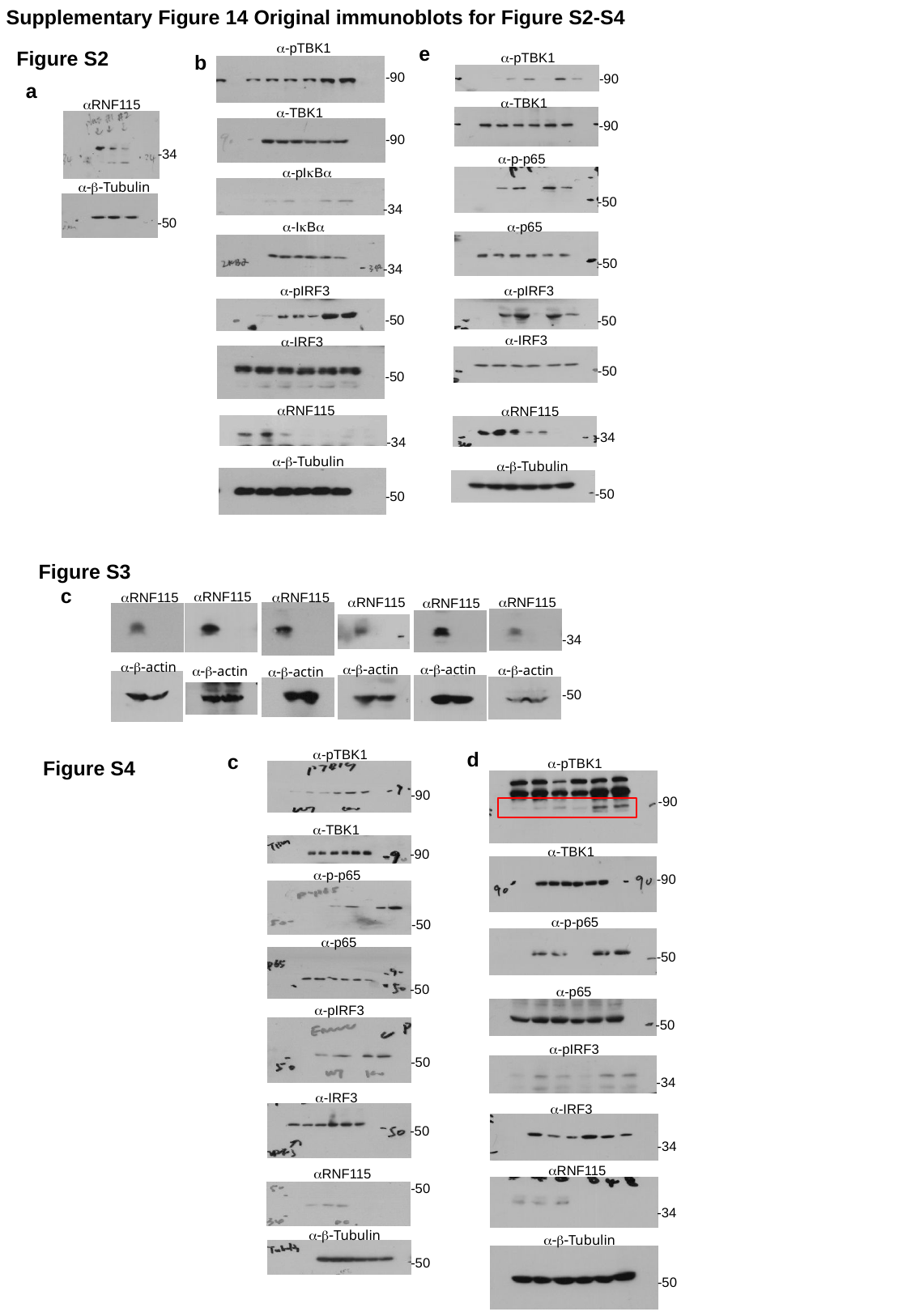

Supplementary Figure 14 Original immunoblots for Figure S2-S4
a-pTBK1
e
 Figure S2
a-pTBK1
b
-90
-90
a
a-TBK1
aRNF115
a-TBK1
-90
-90
-34
a-p-p65
a-pIkBa
a-b-Tubulin
-50
-34
-50
a-p65
a-IkBa
-50
-34
a-pIRF3
a-pIRF3
-50
-50
a-IRF3
a-IRF3
-50
-50
aRNF115
aRNF115
-34
-34
a-b-Tubulin
a-b-Tubulin
-50
-50
 Figure S3
c
aRNF115
aRNF115
aRNF115
aRNF115
aRNF115
aRNF115
-34
a-b-actin
a-b-actin
a-b-actin
a-b-actin
a-b-actin
a-b-actin
-50
a-pTBK1
d
c
a-pTBK1
 Figure S4
-90
-90
a-TBK1
a-TBK1
-90
a-p-p65
-90
a-p-p65
-50
a-p65
-50
-50
a-p65
a-pIRF3
-50
a-pIRF3
-50
-34
a-IRF3
a-IRF3
-50
-34
aRNF115
aRNF115
-50
-34
a-b-Tubulin
a-b-Tubulin
-50
-50

## Slide 7
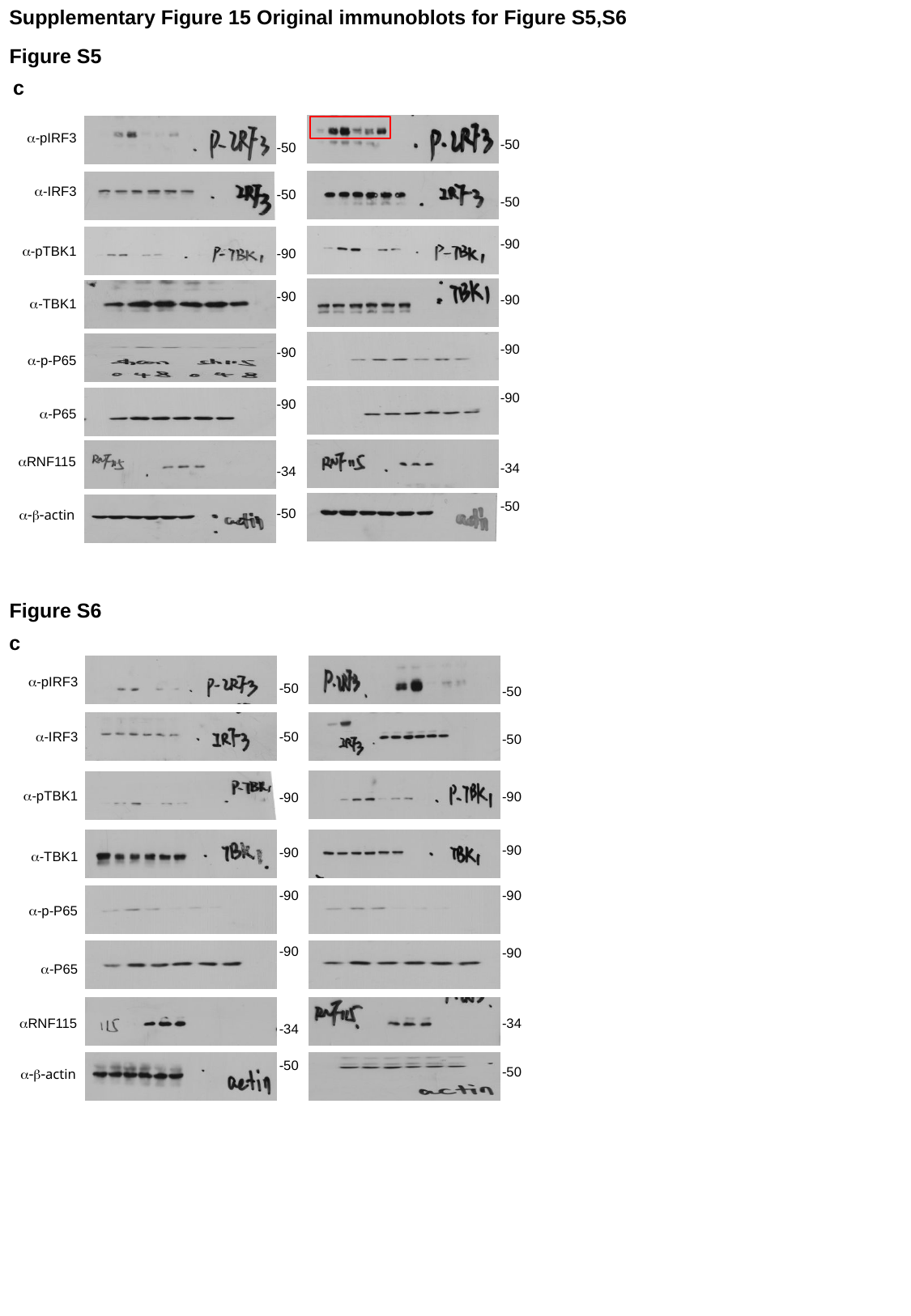

Supplementary Figure 15 Original immunoblots for Figure S5,S6
Figure S5
c
a-pIRF3
-50
-50
a-IRF3
-50
-50
-90
a-pTBK1
-90
-90
-90
a-TBK1
-90
-90
a-p-P65
-90
-90
a-P65
aRNF115
-34
-34
-50
a-b-actin
-50
Figure S6
c
a-pIRF3
-50
-50
a-IRF3
-50
-50
a-pTBK1
-90
-90
-90
-90
a-TBK1
-90
-90
a-p-P65
-90
-90
a-P65
aRNF115
-34
-34
-50
a-b-actin
-50

## Slide 8
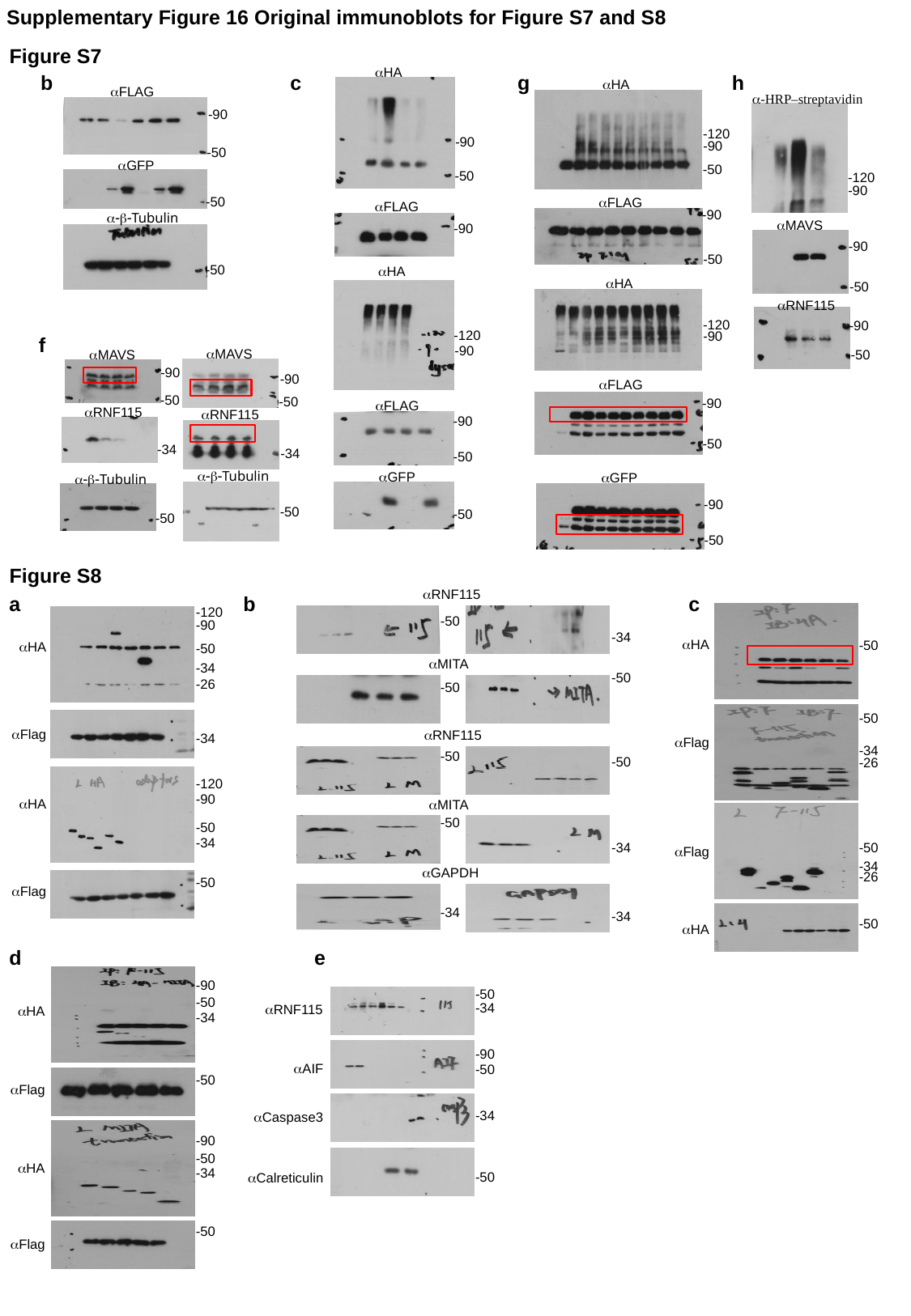

Supplementary Figure 16 Original immunoblots for Figure S7 and S8
Figure S7
aHA
b
c
g
h
aHA
aFLAG
a-HRP–streptavidin
-90
-120
-90
-90
-50
aGFP
-50
-50
-120
-90
-50
aFLAG
aFLAG
a-b-Tubulin
-90
aMAVS
-90
-90
-50
-50
aHA
aHA
-50
aRNF115
-120
-90
-120
-90
f
-90
aMAVS
aMAVS
-50
-90
-90
aFLAG
-50
-50
-90
aFLAG
aRNF115
aRNF115
-90
-50
-34
-34
-50
a-b-Tubulin
a-b-Tubulin
aGFP
aGFP
-90
-50
-50
-50
-50
Figure S8
aRNF115
a
b
c
-120
-50
-90
-34
aHA
-50
aHA
-50
aMITA
-34
-50
-26
-50
-50
aFlag
aRNF115
-34
aFlag
-34
-50
-50
-26
-120
-90
aHA
aMITA
-50
-50
-34
-50
-34
aFlag
-34
aGAPDH
-26
-50
aFlag
-34
-34
-50
aHA
d
e
-90
-50
-50
-34
aRNF115
aHA
-34
-90
aAIF
-50
-50
aFlag
-34
aCaspase3
-90
-50
aHA
-34
-50
aCalreticulin
-50
aFlag
